# Lymphoid‐Tissue‐on‐Chip Recapitulates Human Antibody Responses In Vitro

**DOI:** 10.1002/advs.202521007

**Published:** 2026-04-10

**Authors:** Claudia Teufel, Anna‐Sophie Schlemmer, Andrea W. Eiken, Zachary W. Wagoner, Dennis Vöhringer, Lena Christ, Alex Dulovic, Patrick Marsall, Madeleine Fandrich, Eduardo J. S. Brás, Julia Marzi, Friederike Bärhold, Julia Philipp, Sven Becker, Lisa E. Wagar, Peter Loskill

**Affiliations:** ^1^ Department For Microphysiological Systems Institute of Biomedical Engineering Eberhard Karls University Tübingen Tübingen Germany; ^2^ NMI Natural and Medical Sciences Institute at the University of Tübingen Reutlingen Germany; ^3^ Department of Physiology and Biophysics University of California Irvine California USA; ^4^ Department For Medical Technologies and Regenerative Medicine Institute of Biomedical Engineering Eberhard Karls University Tübingen Tübingen Germany; ^5^ Department of Otolaryngology Head and Neck Surgery University Hospital Tübingen Tübingen Germany; ^6^ 3R Center Tübingen for In Vitro Models and Alternatives to Animal Testing Tübingen Germany

**Keywords:** adaptive immune responses, centrifugal microfluidics, in vitro, lymphoid tissues, NAM, organ‐on‐chips, vaccine research

## Abstract

Reliable modeling of human adaptive immune responses is a prerequisite to understand processes leading to vaccine‐induced protective immunization, to overcome the poor predictive value of non‐clinical in vivo and in vitro models and to drive informed decisions in vaccine development pipelines. Here, we present a centrifugal microfluidics‐based organ‐on‐chip approach to generate an organotypic high density lymphoid‐tissue‐on‐chip. The model enables long‐term culture of lymphoid tissue while preventing autoactivation and shows raised antigen‐specific antibody responses against influenza vaccines for up to 4 weeks on‐chip. Antibody response of different magnitude and quality could be induced both by direct antigen exposure as well as by recruitment of peripheral antigen‐presenting cells. The model represents an attractive approach to evaluate the impact of the mode of antigen delivery on adaptive immune responses. Beyond applications in vaccine development, the lymphoid‐tissue‐on‐chip provides a platform to study cellular interactions during homeostasis, immune responses, and drug treatment over several weeks.

## Graphical abstract

The presented lymphoid‐tissue‐on‐chip system allows culture of primary human tonsil cells at organotypic high density under perfusion for up to 4 weeks, emulates immune response to soluble vaccines and vaccination via peripheral antigen‐presenting cells and represents a useful tool to assess cellular interactions during homeostasis, immune responses, and long‐term treatment with immunomodulators.

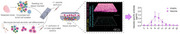

## 1 Introduction

Vaccinations are the best strategy to control endemic and epidemic infectious diseases and to reduce the cumulative impact of infection‐associated morbidities. Through the inclusion of pathogen‐specific target materials, vaccines stimulate an adaptive immune response resulting in the production of antigen‐specific antibodies and T cells. The T cell‐dependent antibody response is a multi‐step process that results in the development of B and T cell memory and long‐lasting antibody responses [1]. This process involves both antigen‐presenting cells from peripheral organs, in particular dendritic cells, as well as stromal cells and immune cells located in secondary lymphoid organs, such as lymph nodes and tonsils [2, 3, 4]. Upon vaccination, peripheral and lymphoid tissue‐resident dendritic cells become activated by adjuvant‐mediated signals through pattern recognition receptors, take up the vaccine antigens, and peripheral dendritic cells migrate to lymphoid organs [5]. In the lymphoid tissues, the interplay of activated dendritic cells, antigen‐specific T follicular helper (Tfh) cells, other antigen‐specific T cells, innate immune cells, and stromal cells promotes an early extrafollicular B cell response and the formation of germinal centers [4, 6, 7, 8]. Direct cell–cell contacts and released factors support B cell survival, proliferation, class switching, and antibody affinity maturation as well as B cell differentiation into germinal center (GC) B cells, memory B cells, short‐lived plasmablasts, and long‐lived plasma cells [3]. Protective antibodies generated during GC reaction are highly specific, neutralize pathogens, and guide immune cell effector functions.

Most of the knowledge on antibody responses originates from in vivo animal studies, predominantly used for non‐clinical vaccine development. However, many vaccines against, e.g., *Mycobacterium tuberculosis* [9] or malaria [10] fail during clinical trials due to poor immunological responses or efficacy within their target population. A retrospective cohort study on the probability of success of vaccines developed against emerging and reemerged viral infectious diseases concluded that only one in ten vaccines progresses from clinical phase II studies to U.S. Food and Drug Administration approval within 10 years [11]. This probability is even reduced to one in 30 vaccines if influenza vaccines produced in well‐established platforms are excluded [11]. Efficacy is also a concern among routinely used vaccines due to its variable protection in different demographic populations, as exemplified in different hepatitis B vaccine response rates in young vs. adult individuals and further confounding factors within the adult population [12]. The poor predictive value of animal vaccine testing originates from described “nonmodifiable discrepancies” between humans and experimental animals, such as inherent differences in the immune system, immunological history, susceptibility to pathogens and pathophysiology, which cannot be overcome by new strategies to increase comparability of data from experimental animals to the human system [13]. Therefore, there is an urgent need for human in vitro models which emulate complex immunological processes, such as vaccine‐induced antibody response.

In recent years, the development of human 2D and 3D lymph node models that reflect functional aspects of central immune responses has undergone significant progress [14]. Models range from simple immune cell co‐culture systems with or without hydrogel, to static and microfluidic culture of lymph node tissue sections and bioreactors and microfluidic platforms [14, 15]. Many of these models recreate individual aspects of immune responses and have proven to be useful to elucidate immune cell migration behavior in signal gradients or the core aspects of GC reaction [15]. However, most of these models rely on blood‐derived immune cells with or without artificial stromal cell lines and therefore lack the complexity of stromal and immune cell heterogeneity necessary to reproduce T cell‐dependent antibody responses. Models with more cellular complexity and advanced spatial microanatomical organization such as lymphoid tissue sections are limited in application due to their short viability of up to 7 days [16, 17, 18]. Furthermore, most models ignore the functional impact of cell density on immune cell function [19]. On the cellular level, the most advanced lymphoid tissue model has been developed by Wagar et al. [20] and consists of tonsil aggregates composed of stromal and immune cells derived from human tonsil biopsies. Tonsil aggregates displayed lymph node‐like spatial cell distribution, mounted antigen‐specific antibody responses against different types of vaccines in a T cell‐dependent manner and recapitulated many characteristics of GC B cell differentiation and antibody maturation, including somatic hypermutation and class switching. Drawbacks are limited culture longevity with a donor‐dependent decline in B cells after 2–3 weeks in culture and the lack of physiological delivery of nutrients, compounds, and particularly antigens, e.g., via peripheral antigen‐presenting cells. In recent years, organ‐on‐chip technology has emerged as a promising tool to generate microphysiological tissue models by modifying the culture environment to meet tissue‐specific cell requirements and by enabling a vasculature‐like perfusion, thereby supporting longevity of various tissue models, enhancing tissue function and allowing for perfusion with different types of immune cells [21, 22, 23].

**FIGURE 1:**
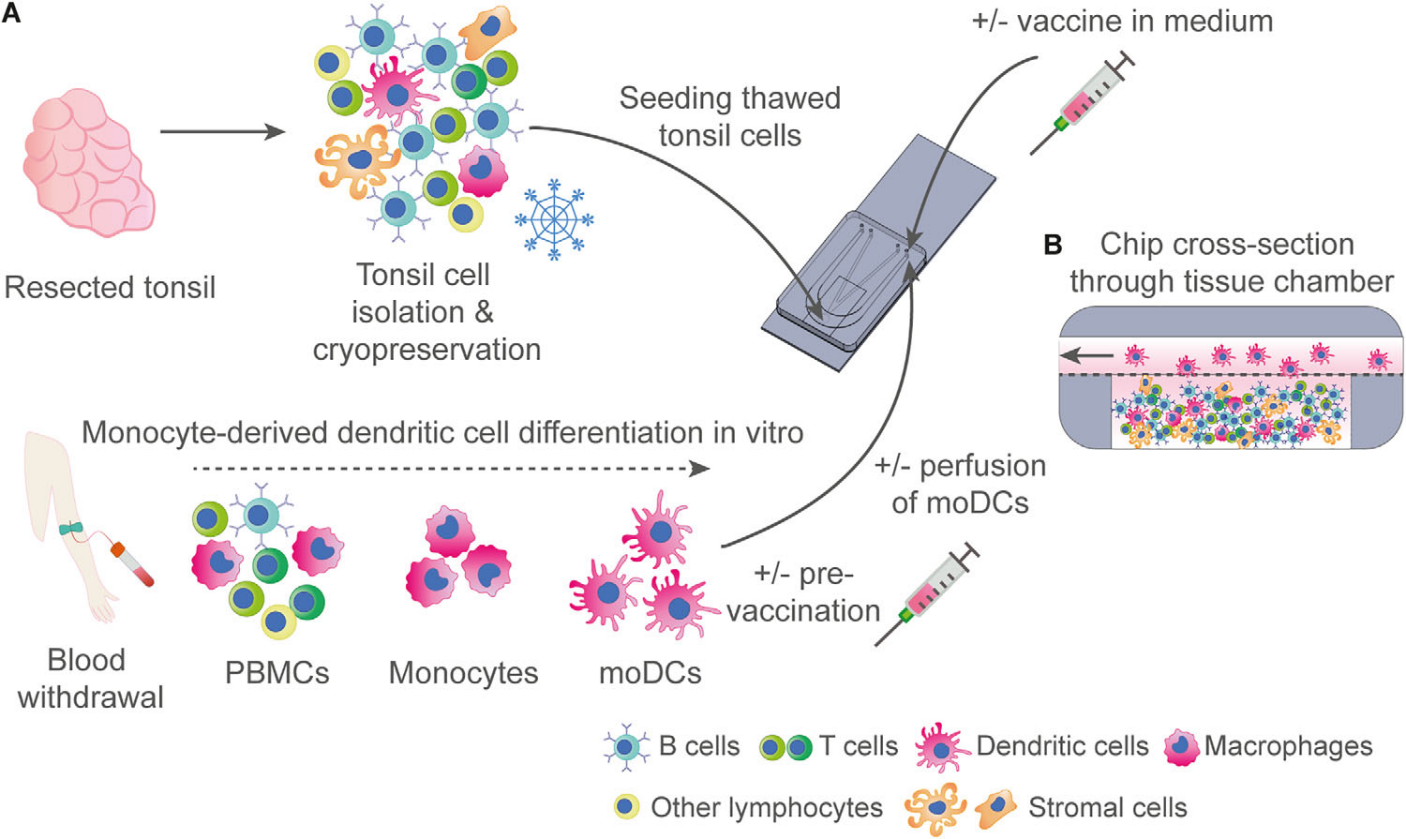
Concept of the lymphoid‐tissue‐on‐chip (LToC) model with adjustable mode of antigen delivery. (A) Schematic of cell isolation, monocyte‐derived dendritic cell (moDC) differentiation from peripheral blood mononuclear cells (PBMCs) and cell component integration into the LToC platform. (B) Schematic of LT chip tissue chamber filled with tonsil‐derived cells and recruitment of perfused moDCs into the tissue chamber.

Here, we merged organ‐on‐chip technology with the tonsil aggregate approach to generate a microfluidic human lymphoid‐tissue‐on‐chip (LToC) model, which enables high‐density culture of lymphoid tissue‐derived immune and stromal cells, increased culture longevity and different modes of antigen delivery (Figure 1). Core features of the model include a specifically designed centrifugal microfluidic lymphoid‐tissue (LT) chip for high‐density lymphoid tissue generation, vasculature‐like perfusion for up to 4 weeks of on‐chip culture and robust assays for vaccination studies highlighting functional antibody‐response upon stimulation directly with antigens or indirectly via autologous antigen‐presenting monocyte‐derived dendritic cells (moDCs).

## 2 Results

### 2.1 Centrifugal Microfluidic Lymphoid‐Tissue Chip Enables Hydrogel‐Free, High‐Density Culture of Tonsil‐Derived Lymphoid Tissue

**FIGURE 2:**
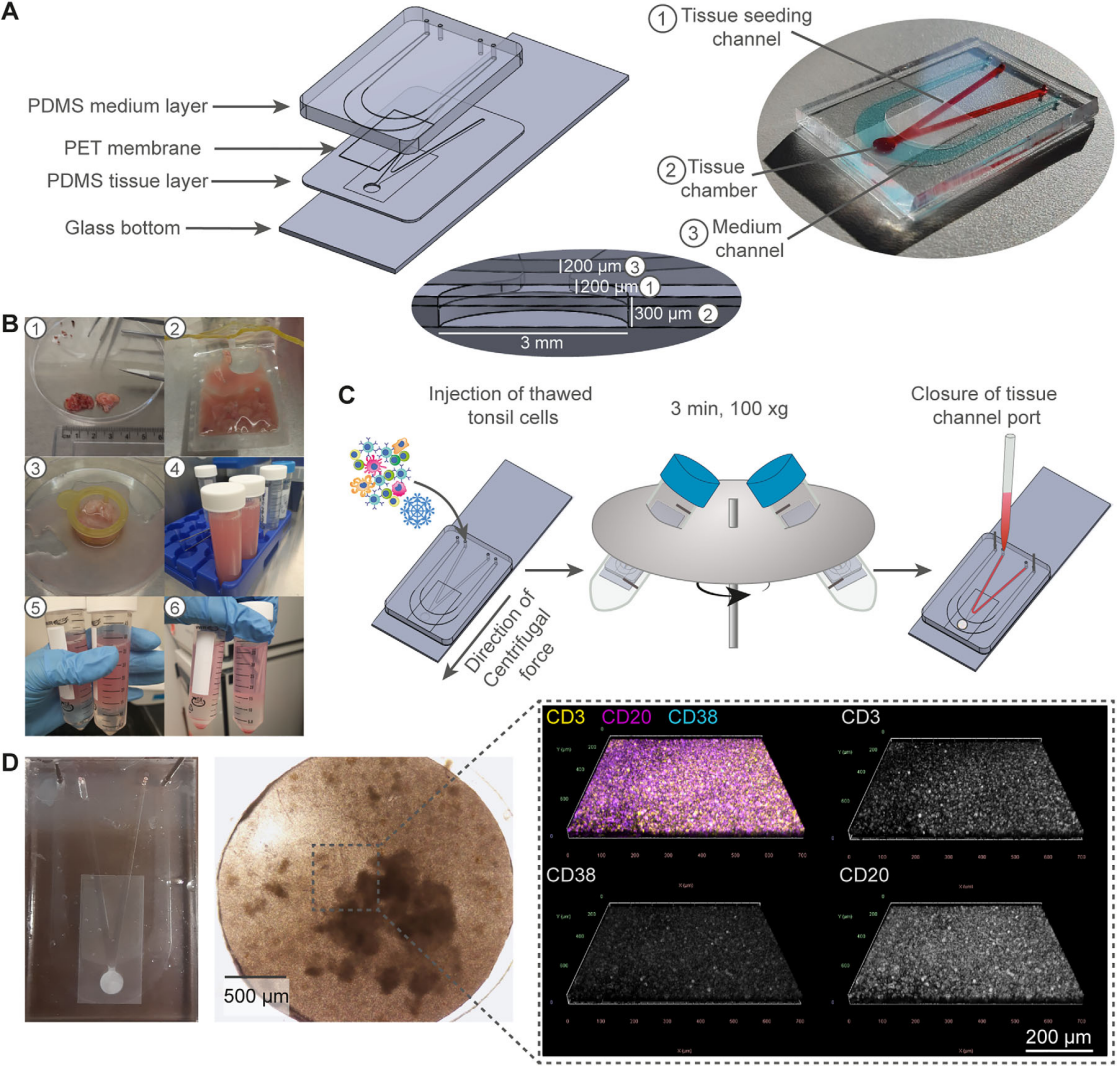
Lymphoid‐Tissue (LT) Chip design, cell injection, and aggregated lymphoid‐tissue‐on‐chip (LToC). (A) Schematic depiction of the four chip layers incorporating polydimethylsiloxane (PDMS) tissue and medium layer, a porous polyethylene terephthalate (PET) membrane and a glass bottom (microscope slide) (left). Dimensions of tissue chamber and medium layers (middle). Watercolor‐filled, assembled LT chip with coverslip glass bottom (right). (B) Images depicting the tonsil isolation process (20): (1) Washed tonsil parts, (2) chopped tonsil fragments after first processing, (3) tonsil fragments after repeated smashing over cell strainer, (4) tonsil suspension before centrifugation, (5) + (6) tonsil cell suspension before and after gradient centrifugation. (C) Schematic of centrifugal cell injection into the LT chip. (D) Exemplary overview image of LToC after 9 days under perfusion (left), bright‐field microscope close‐up image of tissue chamber (middle), and immunofluorescence 3D images of CD20+ B cells, CD3+ T cells, and CD38+ cells in merged and split channel display.

To enable seeding and culture of human lymphoid tissue‐derived cells at high densities, we developed a fit‐for‐purpose polydimethylsiloxane (PDMS)‐based microfluidic LT chip. In the LT chip, cells can be compacted via centrifugation into a tissue chamber (3 mm diameter, 0.3 mm height), which is separated from an overlying medium channel (0.2 mm height) by a porous polyethylene terephthalate (PET) membrane with 5 µm pores (Figure 2A). The incorporated membrane protects the cells from shear stress and is permissive to diffusion of proteins of different sizes, including influenza vaccine hemagglutinin (HA) antigens and human IgG antibodies (Figure S1). The chosen membrane pore size further retains all cells within a confined area upon centrifugation, but at the same time allows for active transmigration of immune cells [22, 24]. Human tonsil biopsies, which are easily accessible and contain all relevant stromal and immune cell subsets involved in T cell‐dependent antibody responses [25], were used as a source for lymphoid tissue‐derived cells and dissociated using a previously established enzyme‐free dissociation protocol [20] (Figure 2B). To generate a LToC, cryopreserved tonsil cells were thawed, injected into the tissue seeding channel of LT chips, and condensed into the tissue chamber by centrifugation (Figure 2C). A total of 3 × 10^6^ tonsil‐derived cells were seeded into each LT chip to ensure the presence of both antigen‐specific B and T cells against individual antigens, which dependent on the antigen, cell type, and cell differentiation status are estimated to be around 1 in 10 000–200 000 cells [26, 27]. The injected cells are estimated to cover the LT chip tissue chamber area with approximately 4.24 × 10^7^ cells/cm^2^ and to generate a compacted, multilayered cell mass of approximately 135 µm height at a cell density of 3.14 × 10^9^ cells/cm^3^ if assuming an average cell diameter of 7.3 µm [28] and a random packing coefficient of 0.64 for spherical particles [29]. After tonsil cell loading into the LT chip, the tissue seeding channel was sealed with Dextran CD hydrogel. The resulting LToC was perfused with medium containing 0.1 µg/mL B‐cell activating factor (BAFF), a B cell survival factor, at 40 µL/h to deliver similar amounts of BAFF as provided in static cultures over 48 h [20]. Upon unidirectional medium perfusion through the medium channel with a syringe pump, tonsil cells populated the tissue chamber in multiple layers and reaggregated into clusters of more condensed regions containing B and T cells (Figure 2D), similar to tonsil aggregates in static plate cultures [20]. Soluble protein signal molecules such as cytokines and human IgG1 antibody were not adsorbed or absorbed onto or into the PDMS‐chips perfused via Tygon tubings (Figure S2). Therefore, the chosen material does not generally interfere with intended measurements of cell‐derived protein signals and antibody release. All in all, the LT chip provides an environment in which a LToC can be generated from tonsil‐derived cells at high density, with continuous medium flow that does not interfere with their capacity for self‐aggregation.

### 2.2 LToC Remains Viable and Maintains Immune Cell Subset Stability Over 4 Weeks of Perfused Culture

**FIGURE 3:**
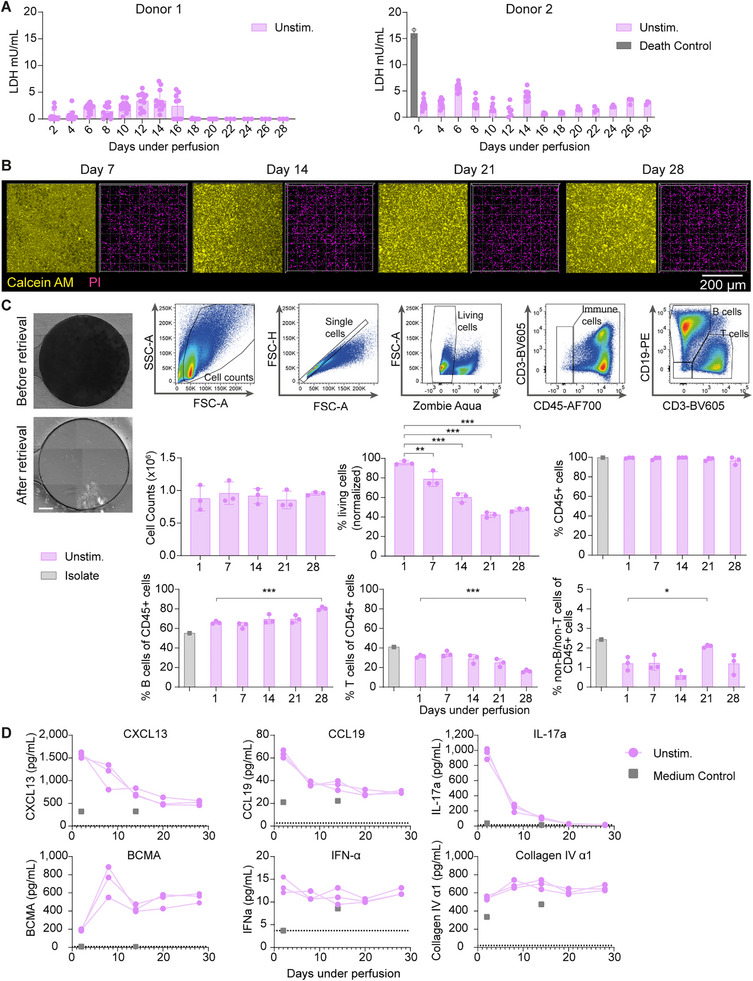
LToC viability, cell subset distribution and protein secretion during 4 weeks of on‐chip culture. (A) Lactatdehydrogenase (LDH) measurements in effluents collected every other day from unstimulated (unstim.) LToCs or cell death control (1% Triton X‐100). *n* = 3–15 chips (unstim.), *n* = 2 chips (death control), error bars represent the average ± SD. (B) Live‐dead‐staining of unstimulated LToC terminated at different timepoints under perfusion. Exemplary 3D top view images of regions within the LToC stained with Calein AM (living cells) and propidium iodide (PI) (dead cells) (Donor 1). (C) Cell retrieval from LToC for longitudinal flow cytometric analysis of cell subset distribution. Images: exemplary LT chip tissue chamber before and after cell retrieval (scale bar 500 µm) and flow cytometry analysis gating strategy. Cell counts estimated from flow cytometry cell counts. Cell viability normalized to initial viability of tonsil isolates. Ratios of immune cells (CD45^+^ cells), B cells (CD19^+^), T cells (CD3^+^), and non‐B/non‐T immune cells in LToC after 1, 7, 14, 21, and 28 days under perfusion and in tonsil isolate before chip loading (isolate). *n* = 3 chips (unstim.) and isolate after thawing (Donor 2). One‐way ANOVA with Dunnet's multiple comparison test was used for comparison against samples measured at day 1 (details in the Experimental Section). *p ≤ 0.05; **p ≤ 0.01; ***p ≤ 0.001. Error bars represent the average ± SD. (D) Multiplex cytokine analysis of LToC effluents and medium control (Donor 2). Effluents were collected every other day and effluents from days 2, 8, 14, and 28 were measured for secretion of CXCL13, CCL19, IL‐17, IFN‐α, B cell maturation antigen (BCMA), and Collagen IV α1. Each continuous line depicts effluents from one LToC. Dashed lines indicate lower limit of quantification. Medium controls were measured from days 2 and 14, representing the two prepared medium batches used during the experiment.

The lifespan of statically cultured tonsil aggregates is limited to about 3 weeks, but the number of recovered living cells and especially naïve B cells already drastically decreases between 2 and 3 weeks of culture for some donors [20]. To determine the longevity of the perfused LToC, we monitored the viability and the immune and stromal cell composition of unstimulated LToC over the course of 4 weeks. Effluent quantification of lactate dehydrogenase (LDH), a surrogate cell death marker, showed largely stable LDH levels over time, indicating that no sudden cell death events occurred throughout the culture period (Figure 3A). Staining with Calcein AM viability dye and propidium iodide cell death counterstaining on weekly sacrificed LToCs showed that large numbers of cells were viable over 4 weeks (Figure 3B). To assess cell viability and to perform immune cell subset analysis, we retrieved cells from LToCs 1 day after seeding and subsequently on a weekly basis, and subjected the retrieved cells to flow cytometric analysis. The absolute number of recovered cells (based on flow cytometry data) was stable over the entire culture period (Figure 3C). Viability of cells retrieved from LToC gradually decreased over the first 3 weeks of culture and stabilized at a viability of about 50% of the initially viable cells after 3 weeks. The percentage of hematopoietic cells (CD45^+^) was maintained around 98%–99% and the overall proportions of B cells (CD19^+^), T cells (CD3^+^), and other non‐B, non‐T immune cells were comparable across all time points, with only a decrease in the frequency of T cells over time. The larger fraction of T cells were T helper cells (CD3^+^CD4^+^) and their percentage increased between 1 day and 1 week after initiation of LToC perfusion (Figure S3). About half of all T helper cells in the LToC were Tfh cells (CD3^+^CD4^+^CXCR5^+^) and the percentage of Tfh cells stayed stable throughout the culture period (Figure S3). Within the stromal cells (CD45^−^), the proportion of blood endothelial cells (BECs, CD31^+^Podoplanin^−^) and lymphatic endothelial cells (LECs, CD31^+^Podoplanin^+^) gradually declined, while the rare fraction of fibroblastic reticular cells (FRCs, CD31^−^Podoplanin^+^CD21^+^CD35^−^) and follicular dendritic cells (FDCs, CD31^−^Podoplanin^+^CD21^+^CD35^+^) remained stable over 4 weeks (Figure S4).

Next, we wanted to determine whether the cells within the LToC preserve functional capacity throughout culture. We therefore collected effluents every second day and applied four exploratory multiplex panels covering a total of 68 lymphoid tissue‐associated cytokines, general immune‐related cytokines, complement components, extracellular matrix proteins, soluble Fas, and soluble cytokine receptor isoforms in LToC effluent collected at five different timepoints to measure secretion and shedding of these analytes by LToC over time (Figure 3D and Figure S5). 29/68 of the measured analytes were below analyte‐specific lower limit of quantification, 3/68 analytes (aggrecan, chemerin, IL‐15) were detectable at equivalent concentrations in LToC effluents and medium control and BAFF, which was supplemented in the medium, was above upper limit of quantification. The concentration of 35/68 analytes in effluent from LToC exceeded medium level concentrations. These included lymphoid tissue‐associated cytokines like CXCL13, CCL19, IL‐17, and interferon (IFN)‐α, immunomodulatory soluble B cell maturation antigen (BCMA) [30] and Collagen IVα1. Most measured analytes were either stable or decreased in parallel to the overall viability of the LToC. The majority of stably expressed analytes comprised chemokines and interleukins, suggesting continuous signaling among tonsil‐derived cells. Only some activation‐ or stress‐related analytes like IL‐1ra, IL‐6R‐α, IL‐8, and IL‐10 were exclusively detected on day 2, which might reflect cellular stress post‐thawing. These findings indicate that the heterogenous population of tonsil cells in the LToC retained the ability to produce lymphoid tissue‐relevant cytokines and other secreted factors for 4 weeks, although particularly inflammatory showed a decline in concentrations over time.

To directly compare if there is a benefit of the LToC over static tonsil aggregate plate cultures, we assessed immune cell subsets in LToC and tonsil aggregate cultures for up to 3 weeks. To account for differences in seeded cell numbers and BAFF concentrations in the two systems, we cultured LToC both at our original setup (3 × 10^6^ cells, 0.1 µg/mL BAFF) and at conditions used in plate cultures (1.5 × 10^6^ cells, 0.5 µg/mL BAFF), and plated 1.5 × 10^6^ tonsil cells with 0.1 µg/mL and 0.5 µg/mL BAFF in ultra‐low attachment plates in parallel. After 3 weeks in culture, the ratio of naive B cells in LToCs seeded at high density was about 50% higher than in LToCs with lower seeding density and tonsil aggregate plate cultures (Figure S6). Reversely, unstimulated tonsil aggregates in plate culture showed about 50% increase in plasmablasts compared to LToC with higher seeding density. In long‐term tonsil aggregate cultures, the percentage of T cells decreased and the ratio of CD4^+^ and CD8^+^ changed, which was not observed in LToC culture (Figure S6). These results indicate a low‐grade autoactivation of tonsil cell aggregate cultures, which is less pronounced in LToC. To test if BAFF concentration might play a role in the improved preservation of the naïve B cell fraction associated with the increased density of the cells within the LToC, we cultured LToC without BAFF, with 0.1 µg/mL BAFF or 0.5 µg/mL BAFF for 3 weeks and analyzed B cell subset ratios at two timepoints. There was no major difference in the ratio of B cells and B cell subsets between these three conditions after 2 and 3 weeks of culture (Figure S7). These findings suggest that the high density within the LToC, which is enabled by the continuous exchange of medium under perfusion, supports the maintenance of non‐activated B cells over longer periods than previously observed and is not strictly dependent on BAFF supplementation.

Taken together, this data demonstrates that the LToC has extended viability compared to other approaches to culture lymphoid tissue, maintains a robust ratio of B and T cells throughout culture, in particular enables long‐term preservation naïve B cells and shows continuous production of many lymphoid tissue‐associated factors.

### 2.3 B Cells Differentiate and Produce Hemagglutinin‐Specific Antibodies On‐Chip in Response to a Single Dose of Quadrivalent Influenza Vaccine

**FIGURE 4:**
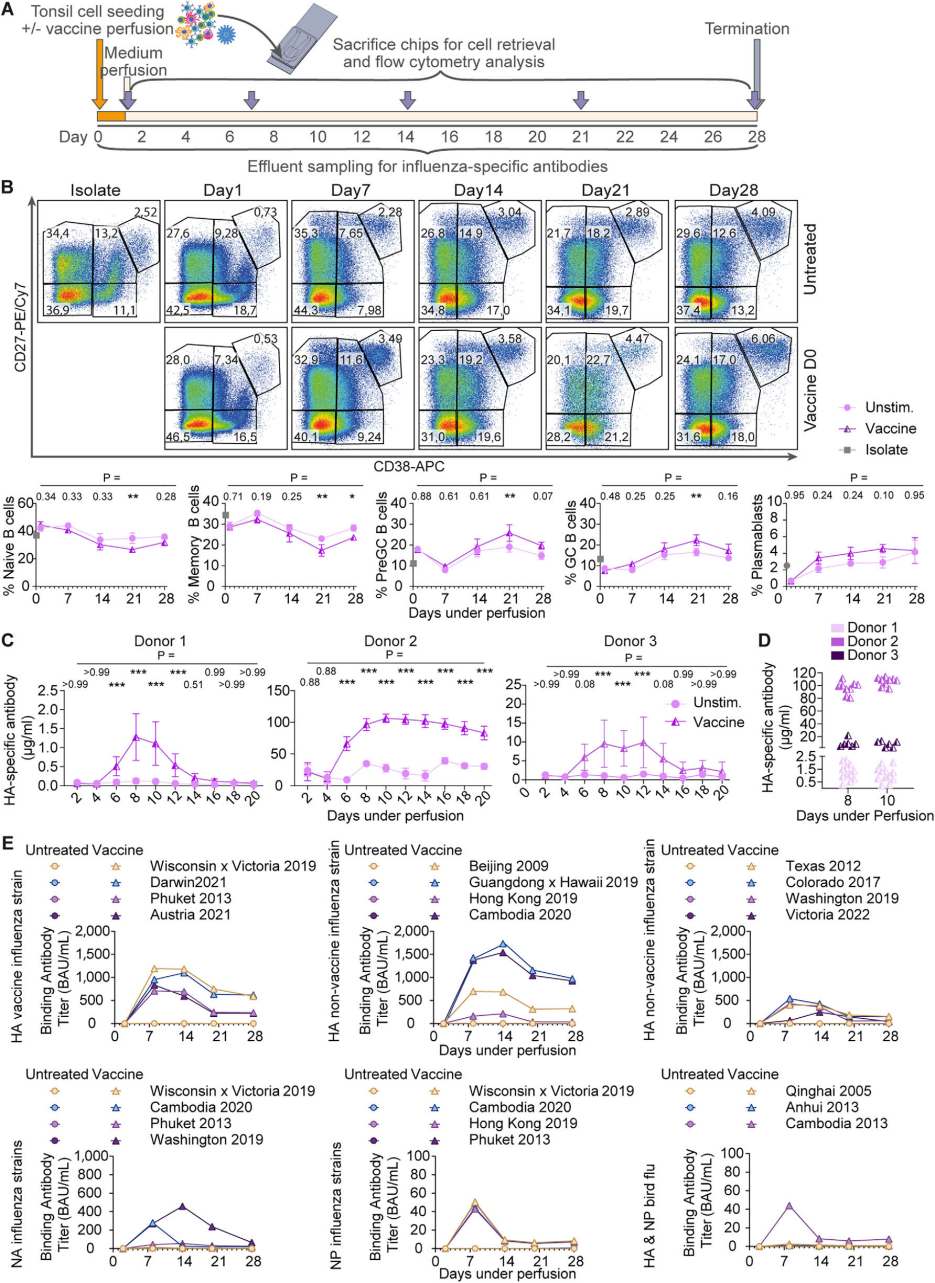
B cell differentiation and antibody production in LToC in response to inactivated split influenza vaccine. (A) Experimental timeline: LToCs were generated and perfused with medium (unstim.) or with vaccine‐supplemented medium (vaccine) for 1 day, followed by perfusion with medium. Effluents were collected every other day and LToCs from each group were terminated for cell retrieval and flow cytometric analysis at different timepoints. (B) Flow cytometric analysis of B cell differentiation status within the B cell (CD19^+^) fraction. Exemplary flow cytometry plots (top) and graphs (bottom) showing percentages of naïve B cells (CD27^−^/CD38^−^), pre‐GC B cells (CD27^−^/CD38^+^), GC B cells (CD27^+^/CD38^+^) plasmablasts (CD27^+^/CD38^+++^), and memory B cells (CD27^+^/CD38^−^) in thawed tonsil cell isolates and throughout LToC culture. *n* = 3 chips per condition (Donor 2). *p* values were determined using two‐way ANOVA with Holm–Šídák's multiple comparisons test (details in the Experimental Section). *p ≤ 0.05; **p ≤ 0.01; ***p ≤ 0.001; *p* values > 0.05 are displayed. Error bars represent the average ± SD. (C) Quantification of influenza HA‐specific antibody release into effluent at different timepoints. *n* = 3–15 chips per condition and donor (three donors). *p* values were determined using mixed‐effect model with Holm–Šídák's multiple comparisons test (details in the Experimental Section). *p ≤ 0.05; **p ≤ 0.01; ***p ≤ 0.001; *p* values > 0.05 are displayed. Error bars represent the average ± SD. (D) HA‐specific antibody quantification in vaccinated chip replicates from Donor 1 (*n* = 12), Donor 2 (*n* = 9), and Donor 3 (*n* = 6). Error bars represent the average ± SD. (E) Multiplex HA subtype‐specific antibody detection was applied to detect binding antibody units (BAU) per mL of antibodies against different influenza strains in effluents from donor 2. *n* = 3 chips per condition (Donor 2), standard deviation not shown.

To evaluate if the LToC model supports GC‐like B cell maturation and antibody responses over extended culture periods, we performed a longitudinal study in which LToCs were exposed to a quadrivalent influenza vaccine (inactivated, split virus; VaxiGrip Tetra 2022/2023) overnight directly after on‐chip seeding and then kept under perfusion for up to 4 weeks (Figure 4A). The B cell differentiation markers CD27 and CD38 were used to track B cell activation and differentiation. While the naïve B cell fraction (CD27^−^CD38^−^) in unstimulated LToCs remained remarkably stable over time, influenza vaccine induced a gradual decrease in naïve B cell proportions, starting approximately 7 days after treatment (Figure 4B). Accordingly, the fraction of pre‐GC (CD27^−^CD38^+^) and GC (CD27^+^CD38^+^) phenotype B cells gradually increased between weeks 1 and 3 of culture. Influenza vaccine further induced B cell differentiation into plasmablasts (CD27^+^CD38^+++^), starting as early as day 7. In line with the phenotypic B cell changes, vaccinated LToC reproducibly produced higher concentrations of influenza HA‐specific antibodies than untreated LToC controls (Figure 4C,D,E). Of note, the kinetics of HA‐specific antibody production of three different donors tested revealed distinct immune regression times, with two donors transiently responding, and one donor producing sustained, high‐magnitude antibody responses from weeks 1 to 4 (Figure 4C,E). Influenza multiplex immunoassay analysis of the effluents from one experiment revealed that IgG antibodies in the effluent from vaccinated LToC bound to HA antigens from the four vaccine influenza H1N1, H3N2, and B strains, but also displayed reactivity against HA antigens from 13 related and unrelated strains and, to a smaller degree, four neuraminidase (NA), and five nucleoprotein (NP) antigens (Figure 4E). The HA‐antibody production rate in the LToC exceeded HA‐antibody production rates in static tonsil aggregate plate cultures from the same donor by up to 90%, even if the seeded cell number and applied BAFF concentrations in the LToC (3 × 10^6^ cells and 0.1 µg/mL BAFF) were adjusted to plate conditions (1.5 × 10^6^ cells, 0.5 µg/mL BAFF) (Figure S8). In summary, the LToC enabled unprecedented retention of naïve B cells over 4 weeks in culture and the observed shifts in B cell differentiation and donor‐specific HA‐specific antibody responses suggest that the LToC model can generate GC‐like antigen‐specific responses to vaccination, rendering the LToC a useful tool to study individual immune responses against vaccines.

### 2.4 LToC Remains Responsive to Vaccination for at Least 3 Weeks

**FIGURE 5:**
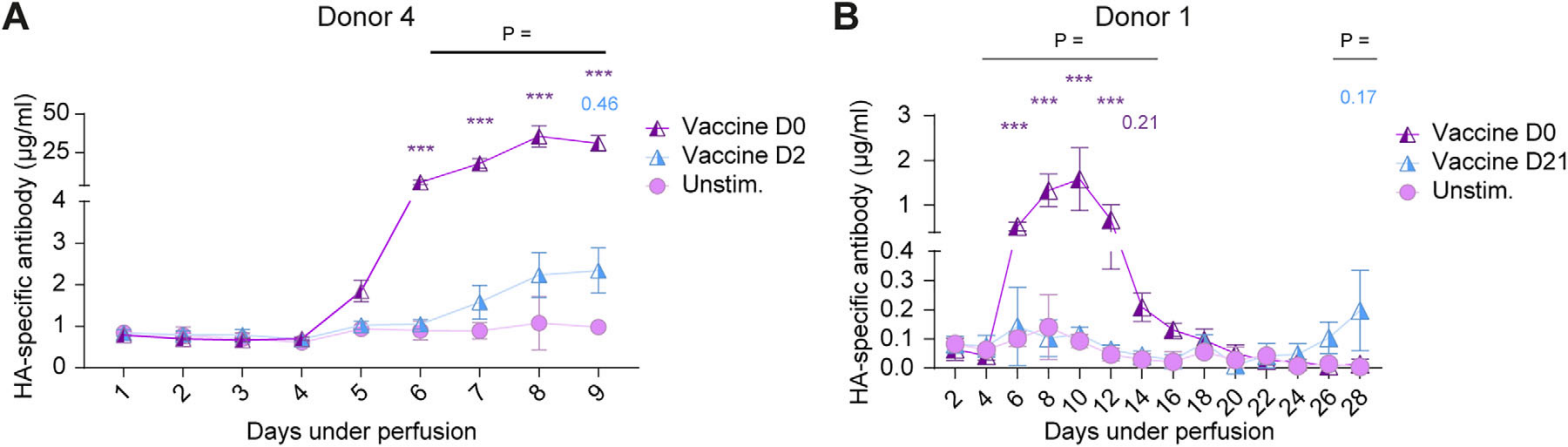
Longitudinal response to inactivated split influenza vaccine. Quantification of influenza HA‐specific antibody release into perfused medium at different timepoints. LToCs were vaccinated at different timepoint of culture and medium was collected daily (A) or every other day (B). (A) unstim.: medium only, vaccine D0: vaccine from day 0 to day 1, vaccine D2: vaccine from day 2 to day 3. *n* = 3–4 chips per condition. (B) unstim.: medium only, vaccine D0: vaccine from day 0 to day 1, vaccine D21: vaccine from day 21 to day 22. *n* = 3 chips per condition. *p* values were calculated using two‐way ANOVA with Dunnett's multiple comparison test (details in the Experimental Section). *p ≤ 0.05; **p ≤ 0.01; ***p ≤ 0.001; *p* values < 0.5 are displayed. Error bars represent the average ± SD.

To determine if untreated LToCs not only survive and persist in a predominantly naïve state, but also retain their ability to functionally respond to vaccine antigens, we next tested their ability to produce an antigen‐specific response when vaccine was introduced shortly after culture initiation (day 0 or 2) or after 3 weeks of LToC culture. To monitor responsiveness, we perfused LToCs overnight with influenza vaccine on days 0, 2, or 21, and monitored HA‐specific antibody secretion for the following week. We observed a gradual, modest increase in HA‐specific antibody production in LToC vaccinated 2 days and 3 weeks after initiation of perfusion (Figure 5). The HA‐specific antibody concentrations in LToC effluents that were vaccinated 2 days and 3 weeks after culture were lower than the HA‐specific antibody concentrations in LToC that received the vaccine dose directly at initiation of LToC perfusion, which might be caused by loss of some HA‐specific B and T cells during culture and decrease of some inflammatory cytokines only secreted immediately after cell loading (Figure 3D and Figure S5). Nevertheless, these findings suggest that the untreated LToC remain functionally capable of low responses to influenza vaccine with antibody secretion over at least 3 weeks of culture.

### 2.5 Peripherally‐Derived Antigen‐Presenting Cells can Migrate Into LToC and Modulate the Extent and Quality of Humoral Responses

**FIGURE 6:**
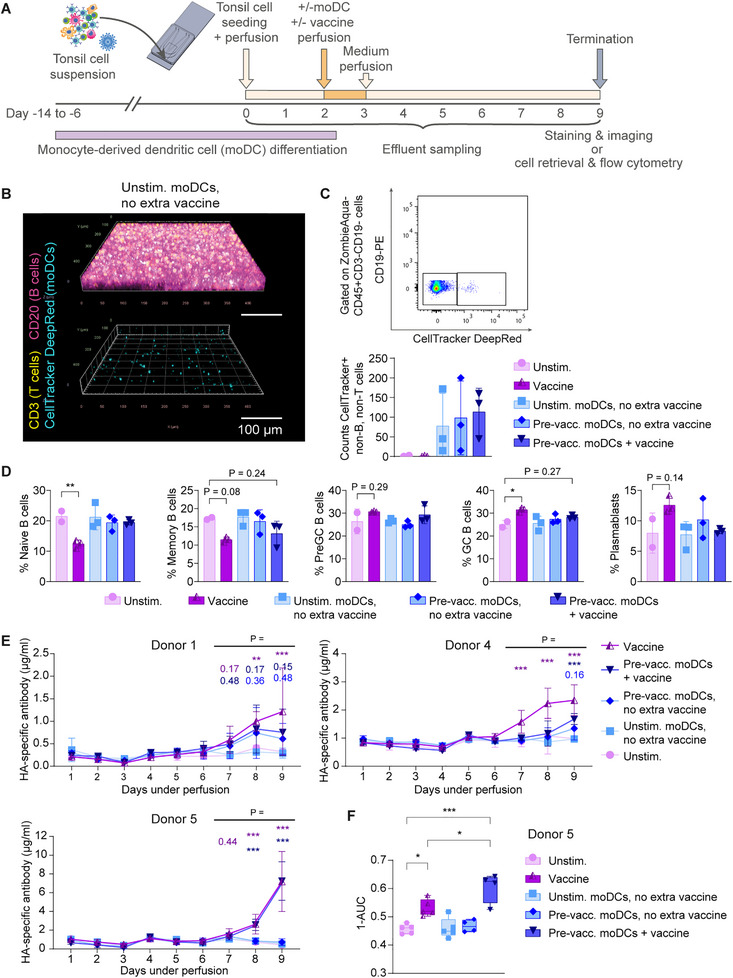
Impact of mode of antigen delivery on B cell responses in LToC. (A) Experimental timeline: Monocyte‐derived dendritic cells (moDCs) were differentiated from thawed autologous peripheral blood‐derived monocytes for at least 6 days and labeled with CellTracker before perfusion. LToCs were generated, perfused with medium for 2 days and then vaccinated overnight by supplementing perfused medium with either vaccine (vaccine), non‐vaccinated moDC (unstim. moDCs, no extra vaccine), moDCs pre‐vaccinated for 3 h (pre‐vacc. moDCs, no extra vaccine), both pre‐vaccinated moDCs and vaccine (pre‐vacc. moDCs + vaccine) or no vaccine (unstim.). Chips were stopped 9 days after perfusion for analysis. (B) Exemplary 3D image of CellTracker‐labeled moDCs, which transmigrated from the medium flow into the LToC (Donor 4). (C) Exemplary flow cytometry plot depicting gating of CellTracker‐labeled moDCs within non‐B non‐T immune cells. Total counts of CellTracker‐labeled non‐B/non‐T cells within cells retrieved from LToC estimated from flow cytometry counts. *n* = 2–3 chips per condition (Donor 5). Error bars represent the average ± SD. (D) Percentages of naïve B cells (CD27^−^/CD38^−^), pre‐GC B cells (CD27^−^/CD38^+^), GC B cells (CD27^+^/CD38^+^), plasmablasts (CD27^+^/CD38^+++^), and memory B cells (CD27^+^/CD38^−^) within B cells determined by flow cytometric analysis. *n* = 2–3 chips per condition (Donor 5). *p* values were calculated using two‐way ANOVA with Dunnett's multiple comparison test (details in the Experimental Section). *p ≤ 0.05; **p ≤ 0.01; ***p ≤ 0.001; *p* values < 0.5 are displayed. Error bars represent the average ± SD. (E) Quantification of influenza HA‐specific antibody release into effluent at different timepoints from three different donors. *n* = 2–5 chips per condition and donor. *p* values were calculated using two‐way ANOVA with Dunnett's multiple comparison test (details in the Experimental Section). *p ≤ 0.05; **p ≤ 0.01; ***p ≤ 0.001; *p* values < 0.5 are displayed. Error bars represent the average ± SD. (F) Microneutralization assay using effluents from LToCs. Quantification by one minus area under the curve (1‐AUC). *p* values were calculated using two‐way ANOVA with Dunnett's multiple comparison test (details in the Experimental Section). *p ≤ 0.05; **p ≤ 0.01; ***p ≤ 0.001; *p* values < 0.5 are displayed. Whiskers indicate the minimum and maximum values and box bottom, middle and top lines represent the first quartile, median, and third quartile, respectively.

Recent evidence suggests that the mode of vaccine administration influences the immune response in secondary lymphoid organs and consequently decides whether a vaccine induces durable and protective memory (31, 32). To determine if the LToC model can be used to study immune response differences depending on the route of vaccine administration, we evaluated if the LToC can recruit antigen‐presenting cells from perfused medium and if the direct addition of vaccine, the indirect addition of vaccine via antigen‐presenting cells, or a combination of both can affect the downstream response in LToC. Therefore, we first isolated monocytes from autologous peripheral blood, differentiated them into antigen‐presenting moDCs, and generated LToCs 2 days before introducing quadrivalent influenza vaccine either through vaccine‐primed moDCs (pre‐vaccinated for 3 h), by direct addition to the LToC culture medium as usual or by combining vaccine‐primed moDCs and direct addition to the medium (Figure 6A). To trace their migration, moDCs were labeled with CellTracker DeepRed. Then we perfused LToC either with non‐primed moDCs, pre‐vaccinated moDCs, pre‐vaccinated moDCs and soluble vaccine, the vaccine only, or left LToC untreated overnight. Seven days after the overnight treatment and continued perfusion with medium, both unstimulated and pre‐vaccinated labeled moDCs were detected within LToC, which demonstrated that the LToC induced active transmigration of moDCs from the medium through the membrane into the tissue chamber (Figure 6B,C). Flow cytometry data from cells retrieved from LToC 7 days after treatment showed that direct vaccination resulted in the previously described drop in naïve and memory B cells as well as the augmented proportions of pre‐GC B cells, GC B cells, and plasmablasts within the B cell compartment compared to untreated LToC, reminiscent of an immune response against influenza vaccine (Figure 6D). Combined treatment with vaccine and pre‐vaccinated moDCs induced similar but less strong trends in B cell differentiation as direct vaccination except for plasmablasts. The observed trends were even lower in LToC which were only perfused with pre‐vaccinated moDCs. As expected, untreated moDCs had no impact on B cell differentiation status in LToC compared to untreated controls. In line with the flow cytometry data, measurements of HA‐specific antibodies in the effluent from LToC revealed that direct vaccination resulted in the strongest antibody responses in two out of three donors, followed by weaker or equivalent antibody responses with combined direct and indirect vaccination, and the lowest but still detectable antibody responses by indirect vaccination alone (Figure 6E). Results from influenza multiplex immunoassay analysis further showed that LToCs vaccinated with a combination of soluble vaccine and pre‐vaccinated moDC produced less IgG antibodies targeting NP and NA influenza antigens and preferentially released IgGs against HA antigens from recent influenza strains (Figure S9).

Finally, we performed a microneutralization assay with the effluent to assess whether the mode of antigen delivery affects the quality of the secreted antibodies. The neutralization capacity of antibodies from LToC that received combined vaccination clearly surpassed the neutralization of capacity of antibodies of LToC that were perfused with vaccine alone (Figure 6F), despite similar magnitudes of HA‐specific antibody (Figure 6E). This suggests that the combination of full antigen and presentation of processed antigens via moDCs improved the quality of humoral responses in the LToC. In summary, these data show that LToC can actively recruit immune cells from the periphery and can be used to determine impact of the mode of antigen delivery on the strength and quality of immune response.

## 3 Discussion

Currently available human lymphoid tissue models are restricted by limited longevity, low cellular complexity, or constraints in the mode of antigen delivery. As a result, studies on the duration of antibody responses, effects of repeated vaccine dosing and further lymph node functions in health and disease have been mostly confined to animal studies. These limitations are addressed by the LToC model reported in this work. The LToCs displayed exceptional long‐term viability and functionality of human lymphoid tissue‐derived cells, supported antigen‐mediated B cell maturation and antibody responses and allowed to discern the magnitude and quality of antibody responses to direct and indirect vaccination modalities. The LToC model showcases how the fusion of appropriate cell sources and new organ‐on‐chip technology can advance modeling of physiological relevant processes towards applications, such as vaccine testing and beyond.

The developed LT chip design ensured injection of highly consistent cell numbers, which is essential for reproducible immune responses between chips. The unique strategy of centrifugal loading introduced a compactness of estimated 3.14 × 10^9^ cells/cm^3^ of the loaded tonsil cells. The microscopically observed cell density in the LToC closely resembled the densities qualitatively observed in native lymphoid tissue [33, 34, 35]. The estimated cell density in the LToC further aligns with the recently reported estimated lymph node cell density of 2–3 × 10^9^ cells/g tissue, which corresponds to 2.06–3.09 × 10^9^ cells/cm^3^ if using the reference‐specific density of tissue of 1.03 g/mL applied in the study [36]. The generated LToCs were marked by a stable cell density as well as stromal and immune cell composition throughout 4 weeks of culture. Appropriate cell density and correct composition of cellular subsets as provided by tonsil‐derived cells is crucial for the preservation of central immune cell functions, as exemplified by the role of MHC self‐recognition [37] and the dendritic cell compartment [38] in maintaining T cell antigen responsiveness in the absence of their cognate antigen in the lymph node. In particular the in vitro maintenance and differentiation of human B cell monocultures requires artificial signals such as addition of CD40 ligand and a mix of cytokines [39] or modified feeder cells [40], which, however, profoundly influence the non‐specific activation and proliferation of B cells. Such activated cultured B cells do not entirely reflect GC processes in vivo and prevent comparisons of treatment effect on B cell subsets within a physiological context. Even in the previously published tonsil aggregates, the ratio of naïve B cells gradually dropped during 3 weeks of culture [20]. In the present work, the naïve B cells fraction was best maintained in LToCs seeded with 3 × 10^6^ tonsil cells when compared to tonsil aggregate plate culture and chips seeded with only 1.5 × 10^6^ cells. This finding argues against donor‐specific loss of B cell inertness in tonsil aggregate plate culture and suggests that the organotypic high density of the LToC supports the preservation of naïve B cells. This observation might indicate either an impaired survival of naïve B cells at lower cell density with less cell‐to‐cell‐contact or a gradual collateral activation through accumulation of cytokines and waste products in static transwell or plate culture. The observed durability of B cells including naïve B cells in LToC was therefore a major advance and enables long‐term interaction studies that require stable unstimulated controls beyond the scope of vaccination and re‐vaccination studies, e.g., immunotoxicological studies that require low background activation in control conditions.

Multiplexed analysis of lymphoid tissue‐associated cytokines, general immune‐related cytokines, complement components, extracellular matrix proteins, soluble Fas, and soluble cytokine receptor isoforms revealed that LToC continuously released lymphoid‐associated, chemotactic cytokines into the effluent including CXCL13 and CCL19, interleukins with chemotactic and immunomodulating functions, and Collagen IVα1. Many of the analytes measured in the effluent were expressed at stable or only slowly declining levels for 4 weeks, while a few quickly decreased after initiation of LToC culture, with some of them reaching medium level by the end of the culture period. The slow decline of some of these secreted factors correlates with the slow reduction in LToC viability and an almost equal loss of cells across all analyzed immune and stromal cell populations. Factors declining quickly or gradually include factors like IL‐17, CCL‐19, and CXCL13, respectively. In secondary lymphoid organs, CXCL13 is majorly produced by follicular dendritic cells [41, 42], but can also be secreted by dendritic cells [42] and Tfh cells [8], while CCL19 is produced by stromal cells [43, 44]. Besides their respective inflammatory and homeostatic functions, cytokines like IL‐17, CCl‐19, and CXCL13 are also associated with the development of secondary and tertiary lymphoid structures [45, 46, 47, 48]. Although we did not confirm the T and B cell organization in our model as demonstrated in the tonsil aggregate system [20] and did not perform in‐depth analysis to follow up on the cell subsets producing the measured factors, the initial peak in factors as IL‐17 and CXCL13 might be indicative of signaling involved in the initial formation of aggregates in tonsil‐derived cultures. The continuous, although reducing secretion of signal molecules such as CXCL13 and CCL19 suggests that LToC supports a persisting homeostatic signaling of remaining stromal and immune cells over time. Most analytes that showed an immediate drop between days 2 and 8 after cell seeding comprised inflammation‐associated factors, e.g., IFN‐γ or IL‐8. These inflammatory factors secreted by unstimulated LToC directly after seeding might have been expressed due to temporary cell stress after cell thawing and chip loading. This assumption is supported by observations that unstimulated cryopreserved blood‐derived immune cells show a transient increase in the expression and release of inflammatory cytokines, such as IL‐1α, IL‐6, or IFN‐γ when compared to freshly cultured immune cells [49]. Nevertheless, the presence of these inflammatory factors at early timepoints may affect the magnitude of immune responses upon stimulation of LToC depending on the timepoint of stimulant application. The differences in the presence of inflammatory cytokines might have contributed to the higher magnitude of antibody production in LToC vaccinated on the day of chip loading when compared to magnitude of antibody responses in LToCs vaccinated 2 days and 3 weeks after chip loading. Another interesting finding is the continuous low‐level expression of Collagen type IV. Collagen type IV has been found along the reticular network of human [50] and non‐human primate [51] lymph nodes, which is crucial for the trafficking of immune cells within the lymph nodes [52]. The Collagen IVα1 found in the LToC effluents implies that the LToC is able to produce own extracellular matrix further enhancing functionality of this model.

The microfluidic LToC system did not impair the previously described self‐aggregation of tonsil‐derived cells [20], as the LToC displayed optically discernable areas of higher and lower density in brightfield imaging even within the compactly seeded tissue chambers, suggesting a similar spatial distribution of cells in LToC and tonsil aggregates. The LToC further allowed retrieval of cells and enabled in‐depth analysis of phenotypic changes of cells during immune responses using flow cytometry. This analysis revealed that B cells from vaccinated LToCs undergo expected GC‐like B cell differentiation processes consistent with the adaptive immune responses observed in the tonsil organoids [20, 53], and therefore reflect features of an in vivo immune response. In our LToC experiments, the observed vaccine‐induced changes in the individual B cell subsets were lower than 10% each. In a small cohort in‐human study by Turner et al. [54], ultrasound‐guided fine needle aspiration (FNA) samples from human lymph nodes at different time points after vaccination with inactivated influenza vaccine showed comparable increase of the percentage of GC B cells ranging from 0.5% to 6% during 1–4 weeks post‐vaccination in vaccination responders. As FNA samples are considered to represent cell populations of excisional biopsies well, their results suggest only low local expansion of antigen‐responsive B cells in draining lymph nodes in response to the vaccine. The low changes in B cell subset ratios in vaccinated LToCs therefore closely reflect early vaccine‐induced changes of B cell populations in humans. In line with the statically cultured tonsil aggregates, but in contrast to a previously published organ‐on‐chip model of ectopic lymphoid follicles built from blood‐derived B and T cells and moDCs [55], vaccination with a quadrivalent influenza vaccine alone induced or re‐activated HA‐specific antibody responses in LToC generated from different patient material. This finding highlights the importance of using the full cell subset diversity found in lymphoid tissue, including stromal cells, to reliably model central immune responses. The magnitude and kinetics of the HA‐specific antibody responses from LToC generated from different tonsil donors varied, which is expected due to the diversity of immune responses among individuals, although screenings of larger numbers of donors are necessary to reflect the full diversity of immune responses. Recent data showed that influenza vaccine responses can be shaped by both host and antigen factors, e.g., the presence of HA‐specific B cell subsets and the frequency of T helper 1 cells [53, 56]. These factors may be influenced by the individual influenza vaccination status and recent influenza infection history, which was unknown for the patients donating biopsy material used during LToC development. Furthermore, all tonsils in the present study were derived from adult participants with ≥ 7 recurrent tonsillitis events per year. The blood of all patients is routinely checked for inflammation markers including C‐reactive protein and abnormal leukocytes counts within basic blood counts before surgery. Although surgeries are only performed in the absence of signs of active inflammation, differences in the inflammatory state of tonsil‐derived cells which persist after cryopreservation cannot be fully excluded. All these factors and differences in the initial proportions of cell types may affect the magnitude of immune responses observed in the LToC system and other systems using tonsil‐derived material. Influenza multiplex immunoassay analysis to determine antibody binding of vaccine‐targeted antigens vs. other influenza antigens showed that vaccination with inactivated split virus led to production of IgG antibodies which bound to all introduced H1N1, H3N2, and influenza B HA antigens, but also showed reactivity to the HA antigens from different strains that occurred between 2012 and 2022. LToCs further generated low antibody responses against influenza NA and NP antigens. This aligns with the finding that individuals vaccinated with different types of inactivated influenza HA vaccines largely raise cross‐reactive or memory HA‐specific antibody responses, but also low‐scale antibody responses against NA and other influenza antigens [57]. Of note, the vaccine‐induced HA‐specific antibody production rate by the LToC vastly exceeded the production rate of tonsil aggregates cultured in plate format generated from the same tonsil cell suspension. This effect could not be attributed to the number of cells seeded into the system alone, suggesting an enhancement of responses due to the organotypic cell density in the LToC. Inactivated quadrivalent influenza vaccine was further applied as well characterized model antigen to test the general responsiveness of the LToC to temporal, specific stimuli at different timepoints. We could show that the LToC remains to some degree responsive to the vaccine even after 3 weeks in culture. The evidence for continuous responsiveness opens the application of the LToC system for studies addressing long‐term immune‐to‐immune cell, host‐to pathogen or immune cell‐to drug interaction in homeostasis, disease, and therapeutic treatments.

Given the role of chemokines such as CXCL13 and CCL19 in immune cell attraction into the lymph node [8, 41, 42, 43, 44], the finding that the LToC produced multiple chemokines suggested that the LToC can attract immune cells from the perfused medium. Indeed, untreated LToC successfully recruited both untreated and pre‐vaccinated moDCs from perfused medium into the tissue chamber. Although the LToC remains a single organ model, we demonstrated that the direct, indirect and combined vaccination strategies elicited immune responses of different magnitude and quality. The strongest effects on B cell differentiation and the highest HA‐specific antibody production were observed when LToCs were directly exposed to the soluble vaccine antigens. Interestingly, the combined delivery of undigested vaccine and pre‐vaccinated moDCs induced lower levels of B cell differentiation, raised lower HA‐specific antibody responses in two out of three donors, produced less influenza NA‐ and NP‐specific IgG antibodies and resulted in production of antibodies with a superior neutralization capacity. This observation indicates that antigen‐specific B cells in the LToC undergo a longer but more efficient and antigen‐targeted antibody maturation due to enhanced T cell help if the antigen is delivered both in processed and unprocessed form. LToCs perfused with pre‐vaccinated moDCs alone did not display observable changes on B phenotypical level, but still mounted a low HA‐specific antibody response. Although HA‐specific B cells require recognition of soluble vaccine to become activated [4, 7], it has been shown that both resident and recently migrated dendritic cells can still display a fraction of unprocessed antigen on their surface and thereby promote T cell‐dependent antibody responses in B cells in the lymph node [58, 59, 60]. Therefore, pre‐vaccinated moDCs might have activated low level B cell responses in the LToC via intact HA proteins on their surface, which were not entirely washed off prior to perfusion. Overall, differences in the magnitude of B cell differentiation and antibody responses dependent on the mode of antigen delivery in our proof‐of concept study demonstrated that the LToC can be used to study immune responses in a more physiological setting and to prevent overestimation of vaccine effect using human in vitro models. Moreover, our findings show that moDCs are not only able to stimulate IgM and cytokine secretion reflecting adaptive immune responses as previously reported for fully blood immune cell‐derived human lymph node models [61], but can also modulate IgG responses in vitro. One limitation remains that we could not apply naturally occurring autologous blood‐derived dendritic cells due to the scarcity of these cells and the low volume of autologous blood that we were able to receive from patients. In a next step, the LToC model would benefit from integration of blood and lymphatic endothelial cell linings in the channel to better model vaccine delivery via lymph and blood and to model the next layer of interaction at the lymphatics and high endothelial venule site of lymphoid organs. This, however, poses specific challenges given the requirement of autologous endothelial cell sources to avoid the development of allogenic reactions against foreign cells in long‐term LToC co‐cultures. A second draw‐back of the LToC system is based on the use of PDMS, which is widely used in the Organ‐on‐Chip field due to its general biocompatibility and ease of use in chip fabrication [62, 63]. Although PDMS did not show adsorption and/or absorption of several tested protein signal molecules and human antibodies, it is known to absorb small hydrophobic molecules [62, 63]. This property can limit the applicability of the LToC model to address some basic research questions and specific drug testing, if input or output molecules of interest exhibit a very low molecular weight combined with high hydrophobicity. Finally, an up‐scaling of the LToC approach is a desirable next step to allow for a higher throughput of the assay. This will allow bigger donor screenings to strengthen conclusions on biological effects that we observed in our newly developed method with only a low number of donors.

To conclude, the LToC model presents a new tool with enhanced longevity to evaluate differences in immune responses to vaccines dependent on the mode of antigen delivery by comparing GC‐like induction of the lymphoid tissue, the quality of antibody responses and the level of cytokine production in perfused medium. Thereby, it can help to determine the efficacy of vaccine candidates in a more physiological setting across groups of individuals of different sex, age, and ethnicity. Beyond the scope of vaccine testing, the LToC model can further be used to study interactions between lymphoid tissue‐derived human cells during homeostasis, immune responses and immunomodulatory therapies over the course of several weeks.

## 4 Experimental Section

### 4.1 Experimental Design

The objective of this study was to develop a microphysiological human lymphoid tissue in vitro model amenable for long‐term culture, which enables replication of human antibody response and antigen delivery via peripheral antigen‐presenting cells. To this end, we designed a fit‐for‐purpose microfluidic Organ‐on‐Chip platform enabling the culture of 3D human lymphoid tissue in a perfused microenvironment. To assess long‐term stability and functionality of the model, LToCs were cultured for up to 4 weeks and challenged with soluble vaccines with or without pre‐vaccinated antigen‐presenting cells and a battery of readouts including flow cytometry, imaging and effluent analysis assays was applied.

### 4.2 Lymphoid‐Tissue Chip Design and Fabrication

Plastic film photomasks (Filmbelichtung24), depicting four tissue and medium layers of the lymphoid‐tissue (LT) chip, were drafted using AutoCAD (Autodesk) and used to fabricate SU‐8 photoresist (Microresist Technologies) features on silicon wafers (Siegert Wafer) via photolithographic processes as described before [22, 64]. As visualized with SolidWorks (Dassault Sytèmes) (Figure 2A), the SU‐8 structures on the tissue layer wafer had three heights: 315 µm for the tissue chamber, 215 µm for the tissue seeding channel, and 15 µm for the membrane inlay (area 9 × 18 mm). Medium channel structures on the medium layer wafer were produced at 200 µm height. Tissue and medium layers were fabricated via replica molding of polydimethylsiloxane (PDMS; Sylgard 184, Dow Corning). PDMS was homogeneously mixed in a 10:1 elastomer base to curing agent mass ratio and degassed in a desiccator. Medium layers were produced using a standard molding approach by pouring the PDMS mixture onto the silicon wafer master mold to obtain PDMS pieces with medium channel structures, followed by curing at 60°C for 4 h. PDMS pieces were cut to the size of the chip and ports were opened using a disposable biopsy punch (diameter 0.75 mm, World Precision Instruments). Tissue layers were exclusion molded by pouring PDMS solution onto the silicon wafer master mold and clamping it against a 5 mm‐thick PMMA disk to produce a layer with through‐hole tissue chamber structures [64]. PDMS was cured at 60°C for 2 h. Before bonding, media layers and isoporous, polyethylene terephthalate (PET) membranes (9 mm × 18 mm, 5 µm pores, 60 000 pores/cm^2^; TRAKETCH PET 5.0 p S210 3 300, SABEU GmbH & Co. KG) featuring an ultra‐thin glass‐like coating (as described previously [64]) were cleaned with isopropanol and blow‐dried with an air pistol and precision wipes (Kimtech Science) if necessary. The microfluidic chips were assembled in three bonding steps: (1) tissue layer to glass coverslip (for imaging, 24 × 40 mm, Knittel) or glass microscope slide (for cell retrieval, 76 × 26 mm, Menzel), (2) coated PET membrane to tissue layer‐glass sandwich, and (3) medium layer to the sandwich from step 2). Bonding was achieved by oxygen plasma activation (75 W, 0.2 sccm O2, Diener Zepto, Diener Electronic) for 24 s before each assembly step, followed by incubation at 60°C for at least 30 min. After step 3, assembled chips were baked at 60°C overnight.

### 4.3 Calculation of Area Cell Coverage, Cell Density, and Cell Mass Porosity in the LToC

For calculations of area cell coverage and cell density in the LToC, an average cell diameter of 7.3 µm (= 3.65 µm) [28] and a random packing coefficient of 0.64 for spherical particles [29] were assumed. LT chambers have a diameter of 3 mm (= 1.5 mm radius)

Cell volume

$$V\;\left(\mathrm{cell}\right)=\frac{4}{3}\;\times \;\pi \;\times \;{\left(0\;000\;365\;\mathrm{cm}\right)}^{3}\approx \;2.04\;\times \;10^{-10\;}cm^{3\;}$$

Total cell volume in chip

$$V\;\left(\mathrm{total}\;\mathrm{cells}\right)=3x10^{6}\mathrm{cells}\;\times \;2.04\;\times \;10^{-10\;}cm^{3\;}\approx \;6.11\;\times \;10^{-4\;}cm^{3\;}$$

$$\begin{array}{ccc} V\;\left(\mathrm{total}\;\mathrm{cells}\;\mathrm{randomly}\;\mathrm{packed}\right) & & =\frac{6.11\;\times \;10^{-4\;}cm^{3\;}}{0.64}\;\\ & & \approx \;9.547\;\times \;10^{-4\;}cm^{3\;} \end{array}$$

Cell layer height in chip

$$A\;\left(\mathrm{tissue}\;\mathrm{chamber}\right)=\pi \;\times \;{\left(0.15\;\mathrm{cm}\right)}^{2}\approx \;7.069\;\times \;10^{-2\;}cm^{2\;}$$

$$\begin{array}{ccc} h\;\left(\mathrm{total}\;\mathrm{cells}\;\mathrm{randomly}\;\mathrm{packed}\right) & & =\frac{9.547\;\times \;10^{-4\;}cm^{3\;}}{7.069\;\times \;10^{-2\;}cm^{2\;}}\;\\ & & \approx \;0.0135\;\mathrm{cm}\;\approx \;135\;\mu m \end{array}$$

Cell density

$$\begin{array}{ccc} \delta \;\left(\mathrm{total}\;\mathrm{cells}\;\mathrm{randomly}\;\mathrm{packed}\right) & & =\frac{3x10^{6}\mathrm{cells}}{9.547\;\times \;10^{-4\;}cm^{3\;}}\;\\ & & \approx \;3.14\;\times \;10^{9}\;\mathrm{cells}/cm^{3\;} \end{array}$$

Cell mass porosity

$$\begin{array}{ccc} & & \phi \;\left(\mathrm{cell}\;\mathrm{mass}\;\mathrm{porosity}\;\mathrm{using}\;V\left(\mathrm{total}\;\mathrm{cells}\right)\right)\\ & & \;=1-\;\frac{V\left(\mathrm{total}\;\mathrm{cells}\right)}{V\left(\mathrm{total}\;\mathrm{cells}\;\mathrm{randomly}\;\mathrm{packed}\right)}\\ & & \;=1-\frac{6.11\;\times \;10^{-4\;}cm^{3\;}}{9.547\;\times \;10^{-4\;}cm^{3\;}}\approx \;0,36\;\approx \;36\% \end{array}$$

Area cell coverage

$$\begin{array}{ccc} \mathrm{Area}\;\mathrm{coverage}\;\left(\mathrm{total}\;\mathrm{cells}\right) & & =\frac{3x10^{6}\mathrm{cells}}{7.069\;\times \;10^{-2\;}cm^{2\;}}\;\\ & & \approx \;4.24\;\times \;10^{7}\;\mathrm{cells}/cm^{2} \end{array}$$

### 4.4 COMSOL Simulations

Numerical simulations were performed using COMSOL Multiphysics 5.5 (COMSOL Inc., Burlington, MA). The Free and Porous Media Flow Module was used to determine the velocity field and shear stress values inside the microfluidic device. Flow in the media channel (free flow area) was governed by the incompressible Navier–Stokes equations, while flow within the cell chamber and across the porous membrane was modeled using the Brinkman equations. Boundary conditions used included the working flow rate of 40 µL/h at the inlet and a 0 Pa pressure value for the outlet while suppressing backflow. A no slip wall boundary condition was also used. Physical properties for the culture medium, such as density and viscosity, were approximated using values for water at 36°C. Porosity of the membrane was obtained from the manufacturer while cell mass porosity in the chambers was estimated as described in the calculation section above (see “Calculation of area cell coverage, cell density, and cell mass porosity in the LToC”).

Transport of Diluted Species Module was employed twice to independently model both the antigen uptake and the antibody release. Convection and mass transfer in porous medium were both enabled to allow a better emulation of the system. Diffusion in both cases was assumed to be isotropic. The diffusion coefficients for the antigen and antibody were calculated using the Einstein–Stokes relation to hydraulic diameter. The hydraulic diameters of IgG and Hemagglutinin have been reported previously [65, 66].

For the antigen uptake, a boundary condition of 0.5 µg/mL was imposed at the inlet and through the coupling with the Free and Porous Media Flow Module, the concentration gradient from the media channel across the tissue chamber was monitored.

For the antibody release, the concentrations measured from Donor 1 were used to calculate a mass flux of antibody release from the cell chamber, while outlet concentration was measured overtime.

### 4.5 Consent and Human Tissue and Blood Sample Collection

One half of each tonsil and blood from six adult donors with recurrent tonsillitis were collected at the Head and Neck Surgery of the University Clinic Tübingen after informed consent as approved by the Ethical Committee of the Eberhard Karls University Tübingen (No. 346/2022BO2). Patient metrics are summarized in Table S1.

### 4.6 Tonsil Cell Isolation and Cryopreservation

Tonsil biopsies were transferred into decontamination solution containing Ham's F12 medium (HyClone, Cytiva) supplemented with 2% FetalClone II Serum (FCS) (HyClone, Cytiva), 2X antibiotic‐antimycotic solution (Gibco), and 1X Normocin (Invivogen) directly after resection and incubated for at least 30 min at 4°C. Tonsil tissue was washed in Ham's F12 medium with 2% FCS and mechanically dissociated as described before [20]. Gradient centrifugation (Histopaque 1077, Sigma‐Aldrich) was applied to remove debris and tonsil‐derived cells were cryopreserved in FCS with 10% dimethyl sulfoxide (DMSO) (PanReac AppliChem) using a CoolCell Cell Freezing Container (Corning).

### 4.7 Isolation and Cryopreservation of Blood Monocyte

Blood was mixed 1:1 with phosphate buffered saline without calcium and magnesium (PBS‐) (PAN‐Biotech) and separated using Histopaque 1077 and SepMate‐50 (IVD) (STEMCELL Technologies) for density centrifugation (1 200 xg, 12 min) within 1–5 h after blood collection. After centrifugation, peripheral blood mononuclear cells (PBMCs) were poured into a fresh tube and washed twice in PBS− supplemented with 0.1% BSA (Sigma) and 2 mM EDTA (Gibco). PBMCs were used directly for monocyte isolation using Pan Monocyte Isolation Kit, human, LS columns, and QuadroMACS (all Miltenyi Biotec) according to manufacturer's protocol. Monocytes were frozen in RPMI 1640 medium (Gibco) with 20% FCS and 10% DMSO using a CoolCell Cell Freezing Container (Corning).

### 4.8 Tonsil Cell Seeding On‐Chip

Chips were sterilized and hydrophilized with O_2_‐plasma (75 W, 0.2 sccm O_2_, Diener Zepto, Diener Electronic) for 5 min. Then channels were entirely flushed through one tissue channel port with 70% ethanol using 200 µL ultrapoint pipette tips (Thermo Fisher Scientific), washed with PBS‐ and stored with PBS‐ (PAN‐Biotech) at room temperature (RT) until use.

Cryopreserved cells were thawed in thaw medium (RPMI 1640 with GlutaMAX supplement (Gibco) with 10% FCS and 1X antibiotic‐antimycotic), resuspended to 3.75 × 10^6^ cells/mL in complete medium (RPMI 1640 with GlutaMAX, 10% FCS, 1X nonessential amino acids (Gibco), 1X sodium pyruvate (Gibco), 1X antibiotic‐antimycotic, 1X Normocin, and 1X Insulin‐Transferrin‐Selen (ITS‐G) (Gibco)) and stored on ice.

For chip loading one tissue channel port was closed with a sterilized metal wire with 0.7 mm diameter (Menzanium). 8 µL of cell suspension (= 3 × 10^6^ cells) was injected into the other tissue channel port and the second tissue channel port and both medium channel ports were closed. Loaded chips were transferred into 50 mL tube (Greiner Bio‐One) with the tissue chamber facing the bottom of the tube and centrifuged at 100 xg for 3 min. After centrifugation, the tissue channel ports were opened, the tissue channel was flushed with 12 µL 3‐D Life Dextran‐CD Hydrogel SG (Cellendes) using 4 mM CD‐linker and the tissue channel ports were closed again. Finally, the medium channel was opened and flushed with 100 µL complete medium by hydrostatic pressure by attaching one empty 200 µL ultrapoint pipette tip at one port and inserting one medium‐filled tip to the other port. The gel was allowed to crosslink at 37°C, 5% CO_2_, and 95% rH for at least 30 min.

### 4.9 Perfused Chip Culture, On‐Chip Vaccination, and Effluent Collection

For chip perfusion, Tygon tubings (0.51 mm inner diameter, Tygon ND 100–80 Medical Tubing, Saint‐Gobain Performance Plastics Pampus GmbH) were connected to a 21 GA stainless steel plastic hub dispensing needle (KDS2112P, Weller Tools GmbH) at one side and to a blunt 21 GA stainless steel needle (detached from the plastic hub by dissolving the glue overnight in a 70% ethanol solution) on the other side and autoclaved. The plastic hub dispensing needle was attached to a syringe (B. Braun) filled with complete medium with 0.1 µg/mL recombinant human BAFF (Biolegend), inserted into a syringe pump (LA‐190, Landgraf Laborsysteme HLL GmbH), and the blunt needle was inserted into one medium channel port. The other port was connected to a blunt 21 GA needle attached to a Tygon tubing inserted into an effluent collection tube. The chips were perfused with 40 µL/h in push mode at 37°C, 5% CO_2_, and 95% rH. For on‐chip vaccination, complete medium with 0.1 µg/mL recombinant human BAFF supplemented with 1:2000 diluted (= 7.5 ng/mL HA per strain) quadrivalent influenza vaccine (inactivated, split virus; VaxiGrip Tetra 2022/2023, 15 µg/mL HA per strain, Sanofi Pasteur) was perfused overnight (∼16 h) and then changed to complete medium with 0.1 µg/mL recombinant human BAFF. Medium was changed every 3–4 days.

Depending on the experiment, effluent samples (= perfused medium) from each chip were collected every day or every other day, centrifuged at 10 000 xg for 3 min to remove debris. For multiplex cytokine assay and antibody measurements samples were stored at −80°C until further analysis. For lactate dehydrogenase assay, effluents were diluted 1:10 in LDH storage buffer (200 mM Tris‐HCl (pH 7.3) (Sigma‐Aldrich), 10% Glycerol (Carl Roth), 1% BSA), and stored at −20 to −80°C.

### 4.10 Tonsil Aggregate Culture and Supernatant Collection

Thawed tonsil cell suspensions used for tonsil cell seeding on‐chip (see above) were plated at 1.5 × 10^6^ cell/well in 200 µL complete medium with 0.1 or 0.5 µg/mL BAFF into 96‐Well Ultra‐Low Binding Flat‐bottom microplates (Corning). 50% medium change was performed every other day by collecting 100 µL supernatant and adding fresh 100 µL complete medium with 0.1 or 0.5 µg/mL BAFF. Collected supernatant was processed and stored for antibody measurements as described for effluents.

### 4.11 In Vitro Differentiation of Monocyte‐Derived Dendritic Cells (moDCs)

Monocyte‐derived dendritic cell differentiation protocol was adapted from previously published protocols [67, 68]. Cryopreserved monocytes were thawed in X‐VIVO 15 medium (Lonza), plated at 2–3 × 10^6^ cells/well into a 6‐well plate (Greiner Bio‐One) in dendritic cell (DC) differentiation medium (X‐Vivo medium supplemented with 10% human serum (Sigma‐Aldrich), 1% penicillin/streptomycin (10 000 U/mL, Pan Biotech), 500 IU/mL GM‐CSF, and 500 IU/mL IL‐4 (both premium grade, Miltenyi Biotec)) and incubated at 37°C, 5% CO_2_, and 95% rH. The next day, the medium was removed and replaced with fresh DC differentiation medium. 50% medium change was performed every 2–3 days until harvesting. Cells were differentiated for a minimum of 6 days.

### 4.12 CellTracker‐Labeling, Pre‐Vaccination, and Perfusion of Monocyte‐Derived Dendritic Cells

Autologous moDCs were harvested, washed in PBS with calcium and magnesium (PBS+), resuspended in 1:1500 (for imaging) or 1:20 000 (for flow cytometry analysis) diluted CellTracker Deep Red (Invitrogen) in PBS+ (Pan Biotech) (= 0.67 µM final concentration) and incubated at 37°C, 5% CO_2_, and 95% rH for 15 min. Cells were then washed and resuspended to 2 × 10^6^ cells/mL in complete medium. Cells were split, mixed 1:1 either with complete medium without (unstimulated moDCs) or with (pre‐vaccinated moDCs) 1 1000 diluted quadrivalent influenza vaccine and incubated at 37°C, 5% CO_2_, and 95% rH. After 3 h of incubation, unstimulated, and pre‐vaccinated moDCs were washed once with complete medium and resuspended to 1 × 10^6^ cells/mL in complete medium with BAFF.

The perfused chip setup was removed from the incubator, the tubing connecting the medium channel to the effluent collection tube was removed and replaced with a 200 µL ultrapoint tip containing 50 µL complete medium with BAFF. Negative pressure was introduced by running the attached syringe pump setting at 40 µL/h in pull mode for at least 30 min 37°C, 5% CO_2_, and 95% rH. Under continued perfusion, 50 µL of untreated moDCs, or pre‐vaccinated moDCs (= 50 000 cells/chip) or complete medium with BAFF was added to the pipette tips of respective chips. For unstimulated LToC, LToC with unstimulated moDCs without extra vaccine and LToC with pre‐vaccinated moDCs without extra vaccine, 350 µL of complete medium with BAFF was added to the respective tips. For LToC conditions with vaccine only or pre‐vaccinated moDCs with vaccine, 350 µL of complete medium with BAFF and 1:2000 diluted vaccine was added. Chips were perfused at 20 µL/h in pull mode overnight (∼16 h) at 37°C, 5% CO_2_, and 95% rH and changed back to perfusion with complete medium with BAFF at 40 µL/h in push mode at 37°C, 5% CO_2_, and 95% rH. Medium was changed once more after 3–4 days of perfusion.

### 4.13 Immunofluorescence Staining On‐Chip

LToC was washed with 100 µL PBS+ through the medium channel by applying hydrostatic pressure using 200 µL ultrapoint pipette tips as described above. LToC was then fixed with ROTI Histofix 4% formaldehyde (Carl Roth) for 15 min or overnight at RT on a rocker (20 rpm, IKA). Then LToCs were flushed once with PBS‐, washed twice with PBS‐ for 15 min on a rocker, blocked with 3% BSA in PBS‐ for 30 min at RT and incubated with 1:25–1:50 diluted CD3 (rabbit, SP162, Abcam), CD20‐PE (human, REA780, Miltenyi Biotec), and optionally with CD38‐APC (mouse, HIT2, Biolegend) antibody in 0.3% BSA in PBS‐at 4°C overnight or for 2 days. LToC was flushed once and washed twice with PBS‐/0.01% Tween 20 (Sigma‐Aldrich) for 15 min on a rocker protected from light. Finally, LToC was incubated with 1:50 diluted rabbit IgG (H+L) cross‐absorbed secondary AF488‐labeled antibody (goat, polyclonal, Invitrogen) in 0.3% BSA in PBS‐ for 3 h to 2 days at 4°C in the dark, flushed once with PBS‐, washed twice with PBS‐ for 15 min and once for 2–3 h on a rocker and stored at 4°C with PBS‐ until image acquisition.

### 4.14 Calcein AM and Propidium Iodide Staining On Chip

LToC was washed with 100 µL PBS+ through the medium channel by applying hydrostatic pressure using 200 µL ultrapoint pipette tips as described above and viable cells were stained with 2 µg/mL Calcein AM Green in PBS+ (Thermo Fisher Scientific) for 1 h at 37°C. Then, propidium iodide was diluted to 50 µg/mL in PBS+ and added for 5 min at 37°C for labeling nuclei of dead cells. Samples were flushed once with PBS+ and washed with PBS+ three times for 15 min on a rocker. Stained chips were fixed with ROTI Histofix 4% formaldehyde overnight at RT on a rocker, washed three times with PBS‐ for 15 min on a rocker and stored at 4°C until image acquisition.

### 4.15 Image Acquisition

Images were obtained using a confocal Laser‐Scanning‐Microscope (LSM 880, Zeiss, Germany). Images were processed by adjusting brightness and contrast using ZEN lite Software (Version 3.4, Zeiss).

### 4.16 Cell Retrieval, Flow Cytometry Staining, and Analysis

For cell retrieval, medium channel ports were closed with metal wires with 0.7 mm diameter and the PDMS layer above the tissue chamber of LToCs was punched out using a 6 mm biopsy puncher (pfm medical). The membrane was carefully lifted with a pipette tip with 100 µL complete medium, cells were resuspended and transferred into cell collection tubes on ice. The medium channel was flushed with 100 µL complete medium once from each port, medium was collected from the tissue chamber and transferred to respective collection tubes. Chips were incubated 20–30 min on ice with 100 µL FACS buffer (3% FCS, 2 mM EDTA, PBS) then, resuspension and medium channel wash steps were repeated with FACS buffer and all suspensions were added to respective collection tubes. Cell collection tubes were then centrifuged, and cells were transferred into 96‐well plate V‐bottom plates for flow cytometry staining in reduced volume. Cells were washed once with FACS buffer, blocked with 1:25 diluted human Fc Block (BD Biosciences) for 10 min on ice and stained for 30 min on ice with live/dead Zombie Aqua Dye (1:200, Biolegend) and 2X antibody cocktail in FACS buffer to reach final concentrations of: CXCR5‐FITC (1:33, J252D4), CD8‐PerCP (1:50, HIT8a), CD38‐APC (1:200, HIT2) for long‐term experiment or CD38‐BV785 (1:100, HIT2) for moDC perfusion experiment, CD45‐AF700 (1:100, HI30), HLA‐DR‐BV711 (1:100, L243), CD3‐BV605 (1:100, OKT3), CD4‐BV650 (1:100, RPA‐T4), CD19‐PE (1:200, HIB19), CD27‐PE/Cy7 (1:100, O323), IgD‐APC/Cy7 (1:50, IA6‐2), CD45‐BV421 (1:100, HI30), CD21‐PE (1:100, Bu32), CD35‐FITC (1:100, 9H3), Podoplanin‐PE/Dazzle594 (1:100, NC‐08), CD31‐BV785 (1:100, WM59), CD3‐APC/Cy7 (1:100, OKT3), CD19‐APC/Cy7 (1:100, HIB19), CD14‐APC/Cy7 (1:100, 63D3), CD16‐APC/Cy7 (1:100, B73.1) (all from Biolegend). Samples were then washed twice with FACS buffer, fixed with 4% PFA (Carl Roth) in PBS‐ for 10 min at RT, washed twice with FACS buffer and stored at 4°C with FACS buffer until measurement with BD LSRFortessa flow cytometer (BD Biosciences). Data were analyzed using FlowJo V10.10 software.

### 4.17 Lactate Dehydrogenase Assay

Lactate dehydrogenase (LDH) release into the media was detected according to the manufacturer's instructions using the LDH‐Glo Cytotoxicity Assay kit (Promega). Luminescence was measured in duplicate in an opaque 384‐well plate (Lumitrac, Greiner Bio‐One) using the Tristar 5 plate reader (Berthold). LDH standard curves to determine LDH activity in each sample in mU/mL were calculated using GraphPad Prism 10.4.0 software and normalized to medium only. Negative values were considered to be below detection limit and plotted as zeros.

### 4.18 Multiplex Cytokine Assay

Cytokines were measured using a commercial custom multiplex human magnetic Luminex assay (R&D Systems, Wiesbaden, Germany, cat no. LXSAHM, see Tables S2 and S3). Effluent samples were analyzed according to manufacturer's protocol. In brief, samples were diluted 1:2 in assay buffer (CD RD6‐52) and pipetted into individual wells of a 96‐well plate. Standards provided by the manufacturer were included on each plate in duplicate. Bead mixes were diluted in diluent buffer (RD2‐1) according to manufacturer's protocol and added to the diluted effluent. Plates were then sealed and incubated on a thermomixer (800 rpm, 21°C) for 2 h, after which unbound sample was removed and beads were washed three times using an automated plate washer (Biotek Multiflo FX). 50 µL biotinylated detection antibody was added to each well and incubated at 800 rpm, 21°C for 1 h. To remove unbound detection antibody, the plates were washed three times, after which 50 µL Streptavidin‐PE conjugate was added to each well and incubated for a further 30 min at 800 rpm, 21°C. The beads were then washed three times, resuspended in 100 µL of wash buffer and analyzed using a FLEXMAP 3D running Luminex xPONENT (v. 4.2) software (Luminex, Austin, USA). Median fluorescence intensity (MFI) values were back‐calculated using a 5PL regression fit to the standard samples (Bio‐Plex Manager, version 6) to determine cytokine concentrations.

### 4.19 Hemagglutinin‐Specific Antibody Detection by Enzyme‐Linked Immunosorbent Assay (ELISA)

ELISA for detection of influenza hemagglutinin‐specific antibodies was performed as previously described [53]. In brief, EIA/RIA high bind microplates (Corning) were coated with 2 µg/mL quadrivalent influenza vaccine (inactivated, split virus; VaxiGrip Tetra 2022/2023, Sanofi Pasteur) in 100 mM sodium carbonate/bicarbonate buffer (Sigma‐Aldrich) overnight at 4°C and blocked with 1% BSA in PBS‐ for 2 h at RT. Effluents were diluted 1:10, 1:50, or 1:100 in PBS‐ and supernatants were diluted 1:100 in PBS‐. Diluted samples were added coated microplates for 1 h at RT. For detection of bound antibodies, horseradish peroxidase‐conjugated anti‐human secondary antibodies to IgM/IgG/IgA (polyclonal, Abcam) were applied for 1 h at RT. Plates were developed to SureBlue TMB substrate (KPL), quenched with sulfuric acid (2N) (PanReac Applichem) and measured at 450 nm using the Tristar 5 plate reader (Berthold). Concentrations of HA‐specific antibodies were estimated using a human monoclonal influenza A hemagglutinin IgG1 antibody (H1N14‐M, alpha diagnostic international) as a standard. For a direct comparison of HA‐specific antibody production in LToC effluents and tonsil aggregate supernatant, the HA‐antibody production rate for time intervals of 48 h (µg/48 h) per LToC and tonsil aggregate well were calculated as follows

For LToC (for consideration of total effluent volume over 48 h)

$$\mathrm{HA}\;\mathrm{specific}\;\mathrm{concentration}\;\mathrm{Day}\;\left(n\right)\frac{\mu g}{\mathrm{mL}}\times \;0.04\frac{\mathrm{mL}}{h}\times \;48\;h$$

For tonsil aggregate culture (for consideration of removal of 50% medium every 48 h and accumulation of produced antibodies in the well)

$$\begin{array}{ccc} & & \mathrm{HA}\;\mathrm{specific}\;\mathrm{concentration}\;\mathrm{Day}\;\left(n\right)\;\frac{\mu g}{\mathrm{mL}}\;\times \;0.2\;\mathrm{mL}\\ & & \;-\;0.5\;\times \;\mathrm{HA}\;\mathrm{specific}\;\mathrm{concentration}\;\mathrm{Day}\;\left(n-2\right)\;\frac{\mu g}{\mathrm{mL}}\;\times \;0.2\;\mathrm{mL} \end{array}$$

with *n* being the respective day of effluent/supernatant sampling.

### 4.20 Multiplex Hemagglutinin Subtype‐Specific Antibody Detection

Various influenza antigens (Table S4) were immobilized on spectrally distinct populations of MAGplex beads using EDC/sNHS coupling as previously described [69]. Following coupling, beads were stored at 4°C, before being combined into a 25x mix prior to use. Effluent samples were thawed, diluted in assay buffer [70] with 25 µL then transferred into individual wells of a 96‐half well plate, after which 25 µL of 1x Bead Mix was added. Samples were then incubated for 2 h at 21°C, 750 rpm, after which unbound antibodies were removed by washing 3x with wash buffer (PBS + 0.05% Tween20). To detect bound IgG antibodies, RPE‐conjugated human IgG (3 µg/mL) was added to each well and incubated at 21°C, 750 rpm for 45 min. Unbound detection antibodies were removed by washing 3x with wash buffer. The beads were then resuspended in 100 µL Wash Buffer, shaken briefly for 3 min at 750 rpm and then measured using an INTELLIFLEX‐DRSE with the following settings: 80 µL sample volume, 50 beads per ID, and a gating range of 7000–17 000. For conversion to BAU/mL, the second International Standard (10/202) was included on each plate, with a 7‐parameter non‐linear regression used to calculate IU/mL. For quality control, intra‐well IgG and gt‐a‐hu‐IgG beads as well as plate control samples were included.

### 4.21 Microneutralization Assay

Microneutralization (MN) assays in this study were conducted with adaptations from previously outlined protocols [71]. Madin–Darby canine kidney cells (MDCKs, ATCC) were cultivated in Eagle's minimum essential medium (EMEM, ATCC) supplemented with 1% antibiotic‐antimycotic and 10% heat‐inactivated FBS and incubated at 37°C with 5% CO_2_. Only early passaged cells were used for MN assays and subculturing of cells occurred upon reaching 80%–85% confluency. One day prior to the assay, MDCK cells were subcultured into flat‐bottomed 96‐well plates at a density of 1.1 × 10^5^ cells/mL in 100 µL (1.1 × 10^4^ cells/well). Organoid culture supernatants were prepared at 100 µL and diluted (1:5) in virus growth media (VGM) consisting of serum‐free EMEM supplemented with 0.6% BSA (Sigma‐Aldrich) and 1 µg/mL *N*‐p‐Tosyl‐l‐phenylalanine chloromethyl ketone (TPCK)‐treated trypsin (Worthington Biochemical) and then serially diluted (twofold) in VGM, 50 µL were left over in all wells. The infectious A/California/07/09 X A/Puerto Rico/8/1934 reassortant H1N1 virus (BEI NR‐44004) was diluted to 50 TCID50 per 50 µL in VGM and subsequently added to the serially diluted supernatants, followed by an incubation period of 1 h at 37°C and 5% CO_2_. Replicate control samples consisting of 100 µL diluted virus only or 100 µL VGM only were also prepared. After incubation, the media from MDCK monolayers was replaced with the serum‐virus mixtures and further incubated for 1 h. Following this, the serum‐virus mixtures were substituted with 200 µL of VGM supplemented with 2% FBS, and cells were incubated for 48 h at 37°C. Post‐incubation, media was removed and cells were fixed in 4% paraformaldehyde (PFA) in PBS for 30 min, washed once in PBS, and then permeabilized in 0.1% PBS/Triton X‐100 (PBS‐T) at room temperature for 15 min. Cells were washed twice with PBS and blocked using 200 µL of blocking buffer (3% BSA) in PBS for 1 h at room temperature. Influenza virus nucleoprotein (NP) was detected utilizing an equal mixture of anti‐NP mAbs (Millipore Cat Nos. MAB 8257 and MAB 8258) diluted (1/1000) in blocking buffer, followed by horseradish peroxidase (HRP)‐conjugated anti‐mouse IgG (KPL) diluted at 1:3000 in blocking buffer. Plates were developed in TMB peroxidase substrate and reactions were halted using 1N HCl. Finally, assays were quantified in an ELISA plate reader at 450 nm using SoftMax Pro 7.1 software. The area under the curve (AUC) of titration curve was determined per condition, followed by 1 minus AUC (1‐AUC) calculation.

### 4.22 Statistical Analysis

Every chip or well is considered an independent biological replicate and every timepoint of analysis as an independent variable. In each run, we performed at least three independent biological replicates for chip experiments and one to two independent biological replicates for plate culture experiments for each timepoint of analysis. Replicates for each donor were performed within one experimental run, and data from different donors originate from individual experimental runs. Tonsil material from donor 1 was used in two individual runs to collect data for the longitudinal study and moDC perfusion experiment. Statistical analysis was performed at sample sizes of *n* ≥ 3. The sample size criteria depended on the logistical complexity of the whole experiment, primary cell availability, availability of pumping systems, and technical issues (e.g., chip loading efficiency and success of perfusion). Chips were randomly allocated into experimental groups after successful loading (loading efficiency 90%–100%) using a numbering system to distinguish them. The numbering system was kept until analysis. Chips were excluded and not analyzed if there were technical issues with the connection to the pumping system resulting in no medium supply of LToC. Data points for all biological replicates with successful perfusion were plotted, except for some flow cytometry data points. Flow data points from successfully perfused chips and wells were excluded due to flow cytometer disturbance during acquisition and were not subject to any other exclusion criteria. For the multiplex assay depicted in Figure S9, only two of the 4–5 available replicates were used due to restrictions on kit availability. In this case, we randomly selected effluents from the first two chips per condition without any pre‐selection based on other data. In longitudinal experiments, the effluent and supernatant of all chips and wells was measured for LDH and HA‐antibody concentrations, respectively. Some chips and wells were discontinued for intermediate analysis of cellular subsets or imaging purposes leading to reduction of *n* numbers in LDH and HA‐antibody measurements over time.

In bar graphs, each dot within each group represents one chip or well. Bar and line graphs represent the average ± SD, unless otherwise stated in the figure caption. The number of independent biological replicates is detailed in each figure caption (*n*).

For longitudinal comparison of unmatched datapoints of viability and cell subsets in unstimulated LToC over time, one‐way ANOVA with Dunnet's multiple comparison was applied and data points from every timepoint were compared against day 1 data points. For longitudinal comparison of retrieved cell subsets in unstimulated and vaccinated LToC groups, chips were considered unmatched samples within each group and two‐way ANOVA with Holm–Šídák multiple comparison was applied. For comparison of HA antibodies in effluents in longitudinal studies and in LToC to plate comparison, measured values at different timepoints from same chips or wells were considered as matched data points. To account for reducing sample numbers over time (= missing values), mixed‐effect model with Holm–Šídák multiple comparison was applied to compare respective untreated and vaccinated groups within each culture condition. Two‐way ANOVA with Dunnett's multiple comparison test was performed for comparison of unmatched data points when multiple groups were compared against unstimulated control in delayed‐vaccination studies and moDC perfusion experiments for both cell subset and HA‐antibody concentration comparison. For comparison of cell subsets in unmatched untreated chips and wells in LToC to plate comparison, the mean of each group was compared with the mean of every other group using two‐way ANOVA with Tukey's multiple comparison.

All statistical analyses were performed using GraphPad Prism 10.4.0 software.

## Funding

Wellcome Leap, Human Organs, Physiology, and Engineering (HOPE) Program, Wellcome Leap, Dynamic Resilience Program, Carl‐Zeiss‐Stiftung.

## Conflicts of Interest

L.E.W. is a coinventor on a patent describing the static immune organoid culture platform (US patent, application no. is 18/094,851). The authors declare no conflicts of interest.

## Supporting information




**Supporting File**: advs75215‐sup‐0001‐SuppMat.docx.

## Data Availability

The data that support the findings of this study are available from the corresponding author upon reasonable request.
