# Supplementary material for: Lymphoid‐Tissue‐on‐Chip Recapitulates Human Antibody Responses In Vitro
Source: Adv Sci (Weinh). 2026 Apr 10:e21007. Online ahead of print. doi: 10.1002/advs.202521007 (PMC13334661; doi:10.1002/advs.202521007)
Supplement: Supplementary file 1 — Supporting File: advs75215‐sup‐0001‐SuppMat.docx. [file ADVS-9999-e21007-s001.docx]

Supporting Information

Lymphoid-tissue-on-chip recapitulates human antibody responses in vitro

Claudia Teufel,^1,2^ Anna-Sophie Schlemmer,^1,2^ Andrea W. Eiken,^1^ Zachary W. Wagoner,^3^ Dennis Vöhringer,^1^ Lena Christ,^2^ Alex Dulovic,^2^ Patrick Marsall,^2^ Madeleine Fandrich,^2^ Eduardo J.S. Brás,^2^ Julia Marzi,^2,4^ Friederike Bärhold,^5^ Julia Philipp,^5^ Sven Becker,^5^ Lisa E. Wagar,^3^ Peter Loskill^1,2,6^*


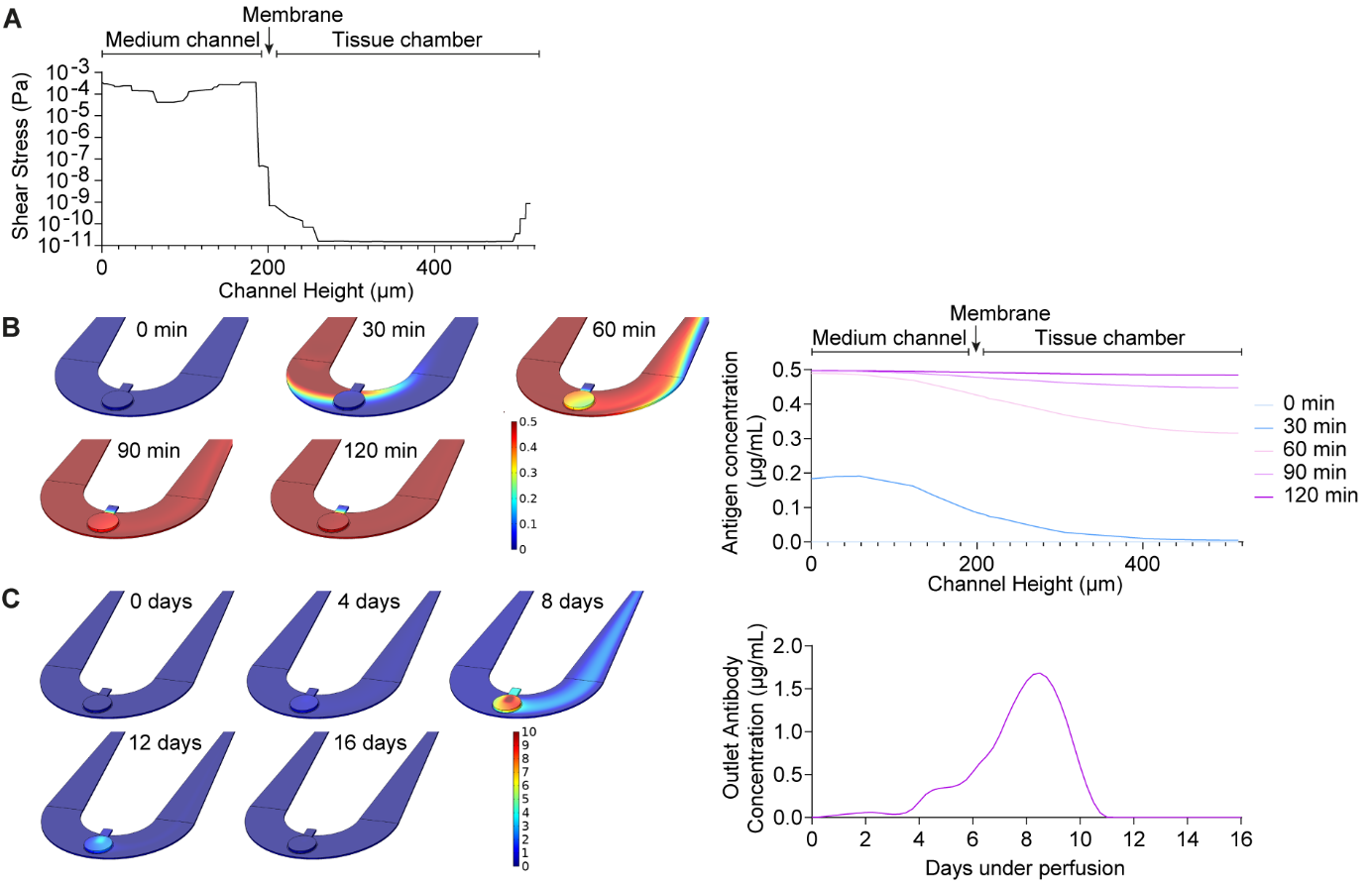


**Figure S1.** COMSOL simulation of shear forces, antigen diffusion and antibody diffusion in the LToC system. (A) Shear forces across the LToC calculated at the center of the tissue chamber. (B) Diffusion kinetics of influenza vaccine hemagglutinin (HA) antigen into the LToC within one hour. (C) Diffusion kinetics of LToC-secreted antibodies into effluent simulated for donor 1. A flow rate of 40 µL/h as applied in the LToC was used in all simulations.


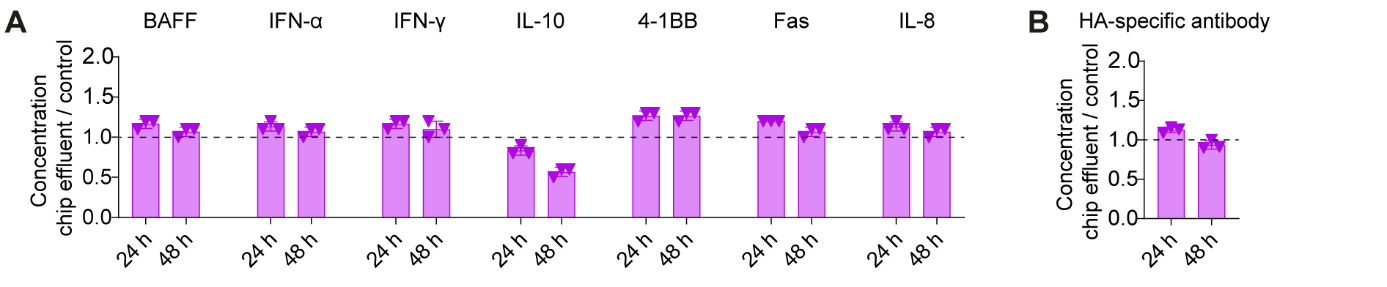


**Figure S2.** Characterization of cytokine, soluble protein signal molecule and antibody adsorption/absorption in the perfused LT chip. To test adsorption and absorption of cytokines, other soluble protein signal molecules and human IgG to the plastic material parts of the LToC system, the standard of a human magnetic Luminex assay and a human monoclonal influenza A hemagglutinin IgG1 antibody were diluted 1:10 and to 0.1 µg/mL in complete medium, respectively. The resulting input standard solution was perfused through empty LT chips via syringe pumps and Tygon tubings of same length as in the LToC setup at 37°C, 5% CO2 and 95% rH. Effluents were collected 24 h and 48 h after start of perfusion. To define the magnitude of adsorption/absorption, an aliquot of the input standard solution (=control) was kept at 37°C, 5% CO2 and 95% rH for corresponding periods of time to account for individual protein analyte instability at 37 °C. (A) Ratio of respective protein concentrations measured in chip effluent and input standard solution at 24 h and 48 h after start of perfusion. (B) Ratio of human monoclonal influenza A hemagglutinin IgG1 antibody concentration measured in chip effluent and input standard solution. n = 3 chips, dashed lines indicate ratio at equal concentrations in effluents and input standard solution.


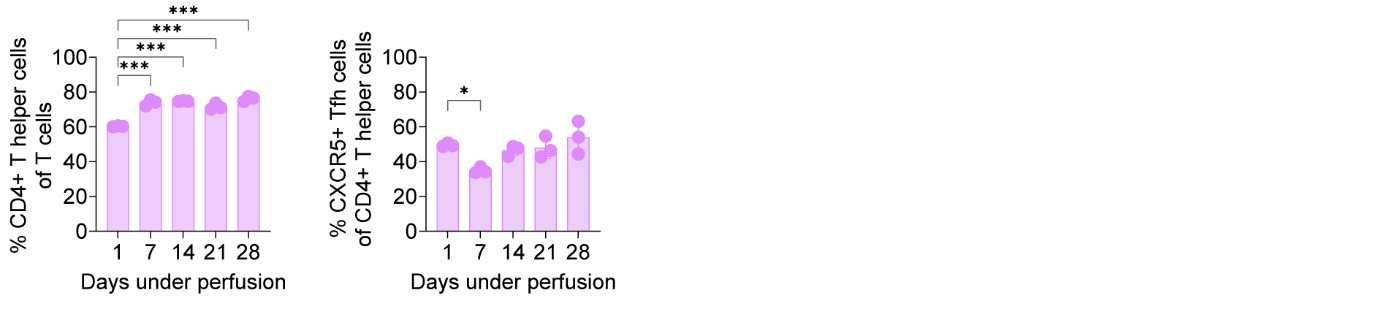


**Figure S3.** Longitudinal analysis of T helper cells in LToC. Cells were retrieved from unstimulated LToCs for longitudinal flow cytometric analysis of cell subset distribution. Ratio T helper cells (CD3^+^CD4^+^) within T cell (CD3^+^) and percentage of Tfh (CD3^+^CD4^+^CXCR5^+^) within T cells are displayed. n = 3 chips (Donor 2). One-way ANOVA with Dunnet’s multiple comparison test was used for comparison against samples measured at day 1 (details in methods section). *P ≤ 0.05; **P ≤ 0.01; ***P ≤ 0.001. Error bars represent the average ± SD.


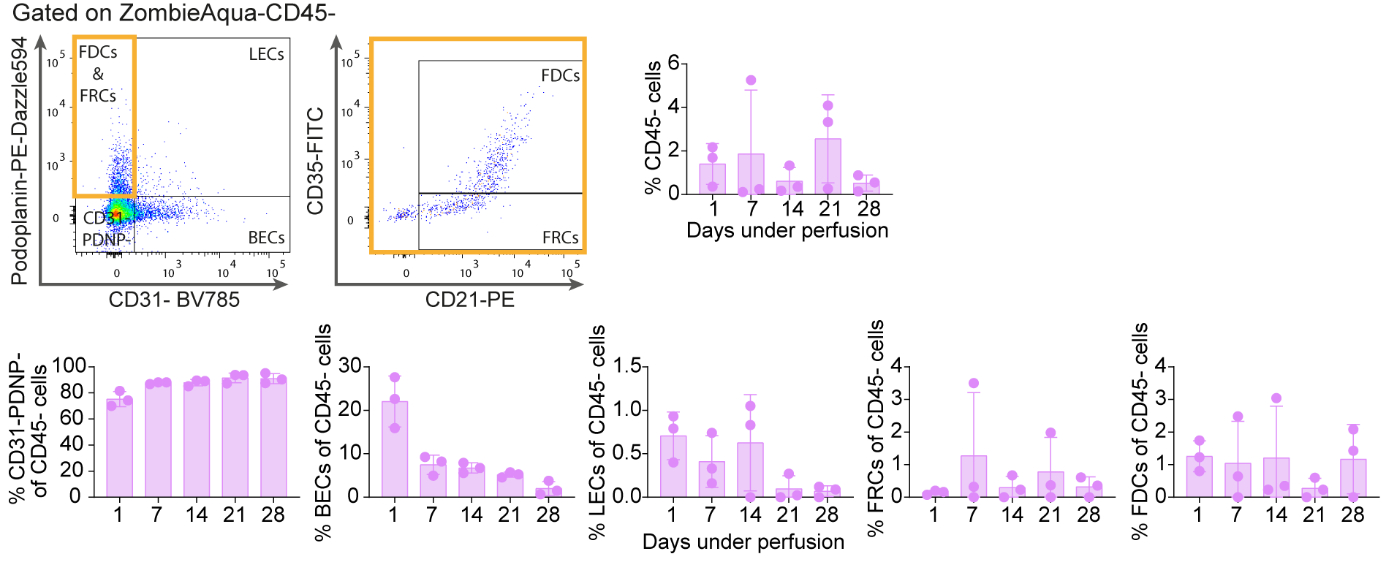


**Figure S4.** Longitudinal analysis of stromal cells in LToC. Cells were retrieved from LToC and assessed for stromal subsets by flow cytometry at different timepoint. Ratios of stromal cells (CD45^-^ cells), unspecified stromal cells (CD31^-^Podoplanin^-^), blood endothelial cells (BECs, CD31^+^Podoplanin^-^), lymphatic endothelial cells (LECs, CD31^+^Podoplanin^+^), fibroblastic reticular cells (FRCs, CD31^-^Podoplanin^+^CD21^+^CD35^-^) and follicular dendritic cells (FDCs, CD31^-^Podoplanin^+^CD21^+^CD35^+^) after 1, 7, 14, 21 and 28 days under perfusion. n = 3 chips (Donor 2).


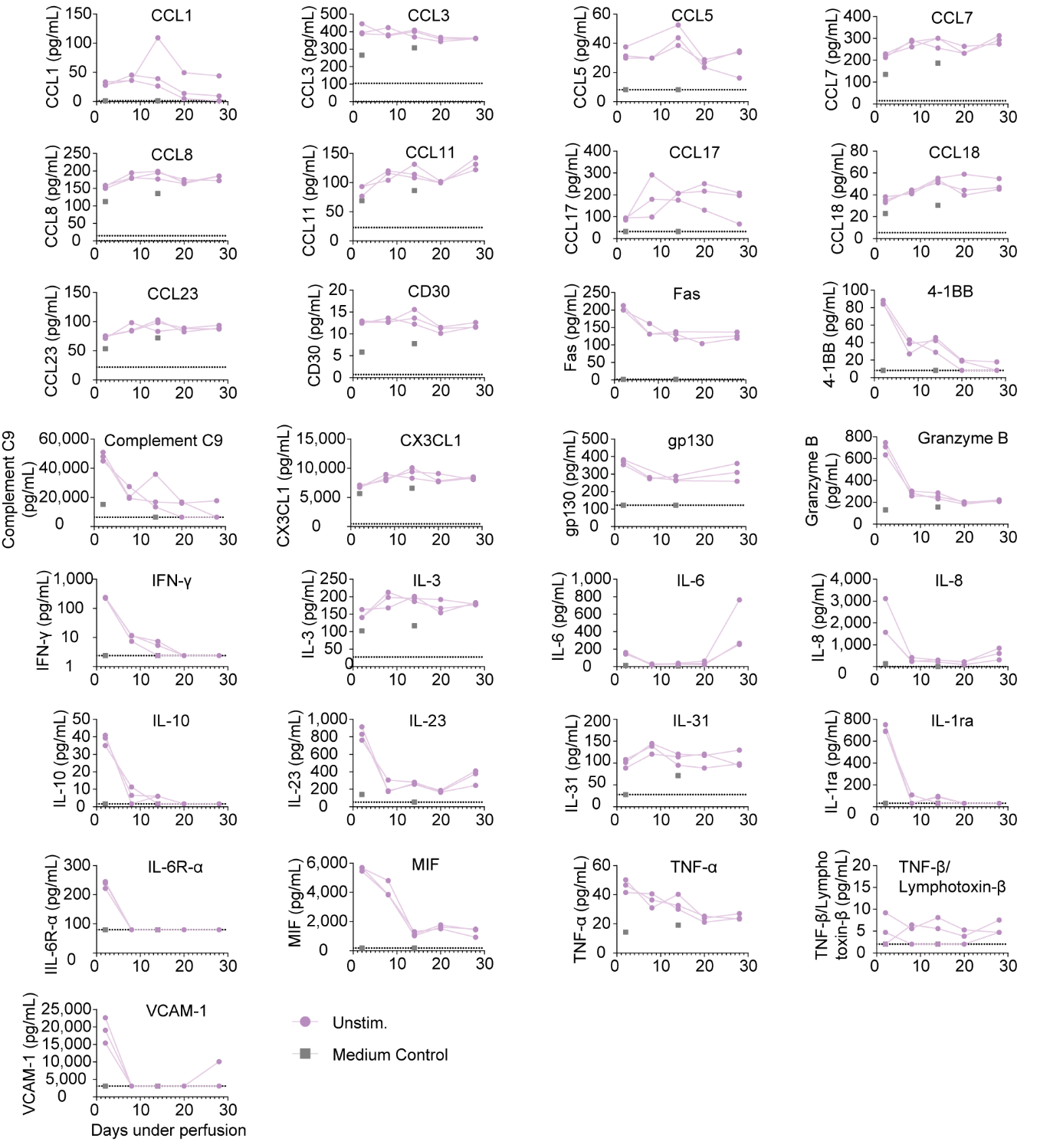


**Figure S5.** Cytokines and other signal molecules in LToC. Multiplex cytokine analysis of LToC effluents (unstim.) and medium control. Effluents were collected every other day and effluents from day 2, 8, 14 and 28 were measured for secretion of respective analytes. Each continuous line depicts effluents from one LToC. Dashed lines indicate detection limit of respective analyte. Medium controls were measured from day 2 and day 14, representing the two prepared medium batches used during the experiment (Donor 2).


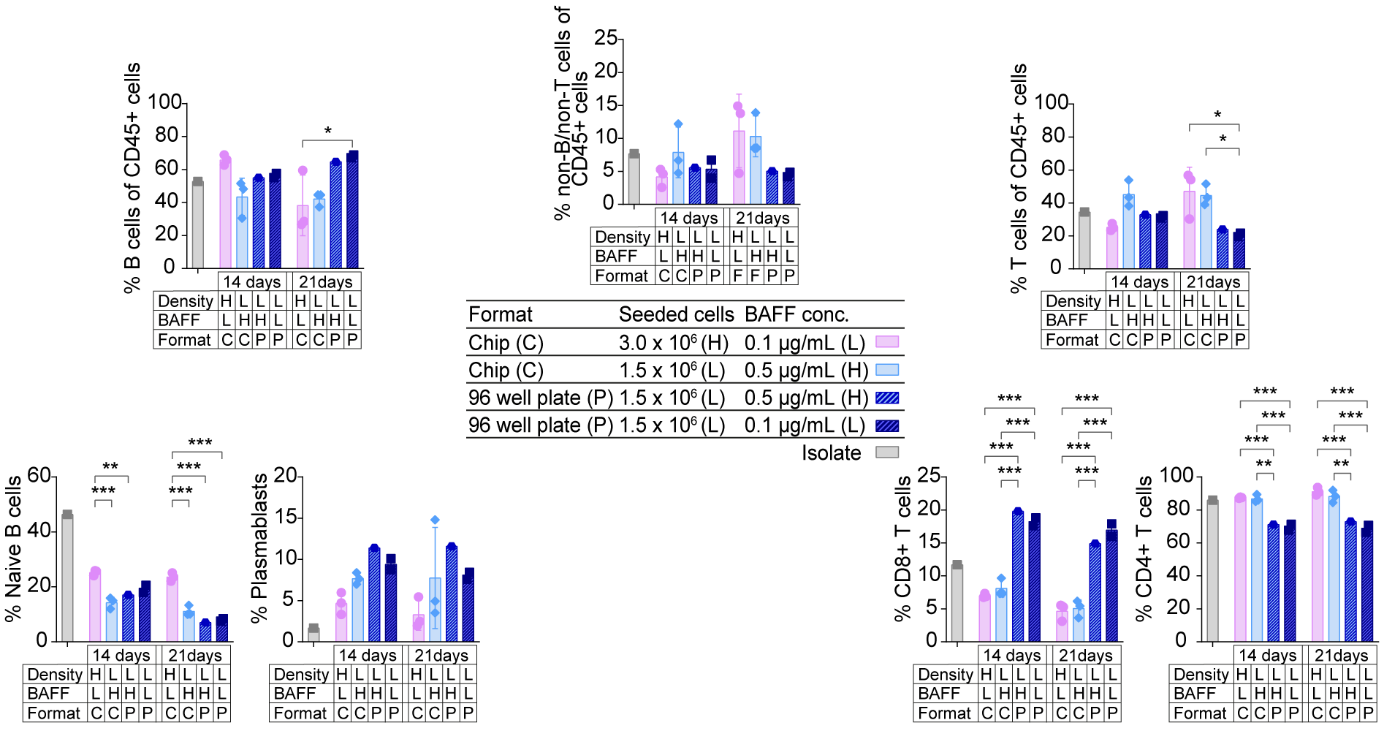


**Figure** **S6.** Differences in cell naivety and distribution in LToC and static tonsil aggregate plate culture with different seeding cell numbers and BAFF concentrations. Tonsil cells were cultured in LToC with original conditions (3 x 10^6^ cells, 0.1 μg/mL BAFF) and at conditions used in plate cultures (1.5 x 10^6^ cells, 0.5 μg/mL BAFF) and plated at 1.5 x 10^6^ tonsil cells/well with 0.1 μg/mL and 0.5 μg/mL BAFF in ultra-low attachment plates. LToCs and plate cultures were stopped at different timepoints and cell subsets were analyzed for B cells, T cells and non-B non-T immune cells within CD45^+^ immune cells as well as B and T cell subsets. Only subsets with discernable differences between conditions are displayed. n = 2-3 for chips and n = 1-2 wells for plate cultures (Donor 3). To determine P values, every group was compared against every other group using two-way ANOVA with Tukey’s multiple comparison (further details in methods section). *P ≤ 0.05; **P ≤ 0.01; ***P ≤ 0.001.


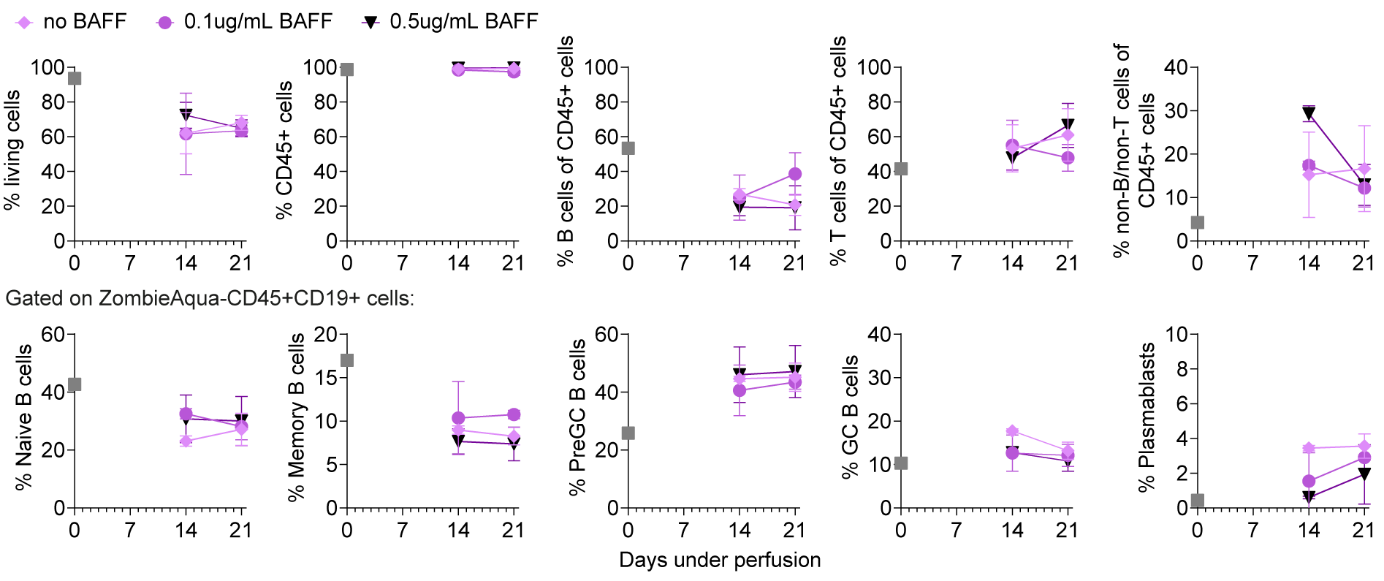


**Figure S7.** B cell subset distribution in LToC with different BAFF concentrations. 3 x 10^6^ tonsil cells were cultured in LToC with different BAFF concentration in perfused medium. LToCs were stopped at different timepoints, and cell subsets were analyzed for B cells, T cells and non-B non-T immune cells within CD45^+^ immune cells. n = 2-3 chips per condition (Donor 6).


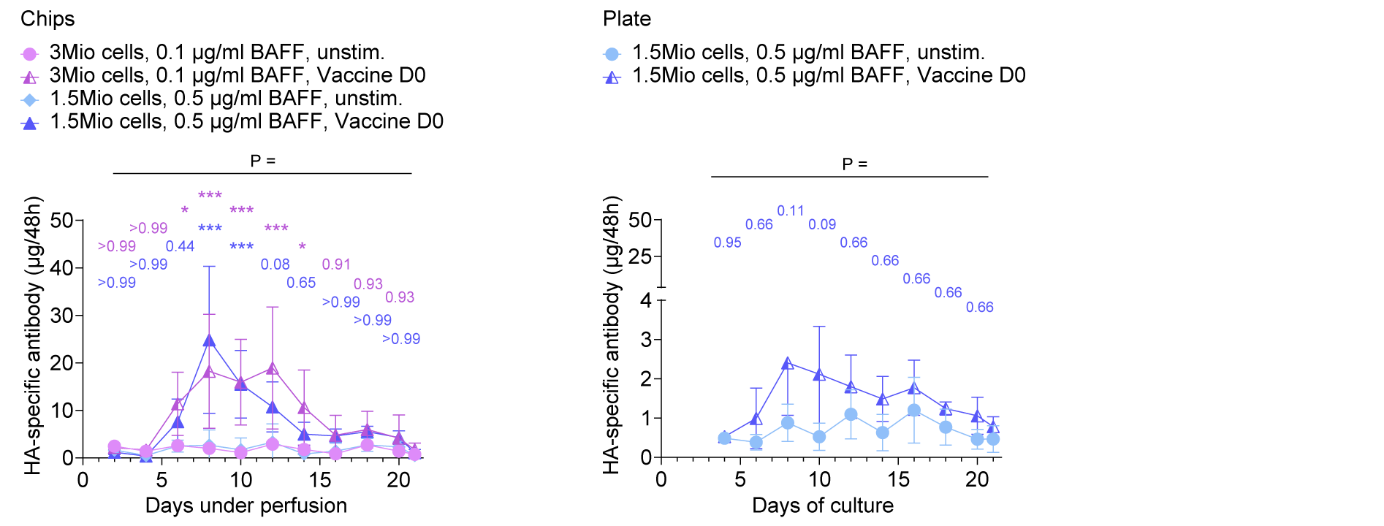


**Figure S8.** HA-antibody production in LToC and tonsil aggregate plate culture. Tonsil cells were cultured in LToC with original conditions (3 x 10^6^ cells, 0.1 μg/mL BAFF) and at conditions used in plate cultures (1.5 x 10^6^ cells, 0.5 μg/mL BAFF) and plated at 1.5 x 10^6^ tonsil cells/well with 0.5 μg/mL BAFF in ultra-low attachment plates. LToC and wells were left untreated (unstim.) or received one vaccination dose at the day of cell seeding (Vaccine D0). LToC effluents and tonsil aggregate supernatant were collected every other day and influenza hemagglutinin (HA)-specific antibody release into effluents was analyzed. To compare antibody production in LToC effluents and plate supernatants, the HA-specific antibody production rate per 48 h (µg/48 h) was calculated from the measured HA-specific antibody concentrations as described in the methods section. n = 3-6 for chips and n = 2-4 wells for plate cultures (Donor 3). To determine P values, mixed-effect model with Holm-Šídák multiple comparison was applied to compare respective untreated and vaccinated groups within each culture condition (further details in methods section). *P ≤ 0.05; **P ≤ 0.01; ***P ≤ 0.001.


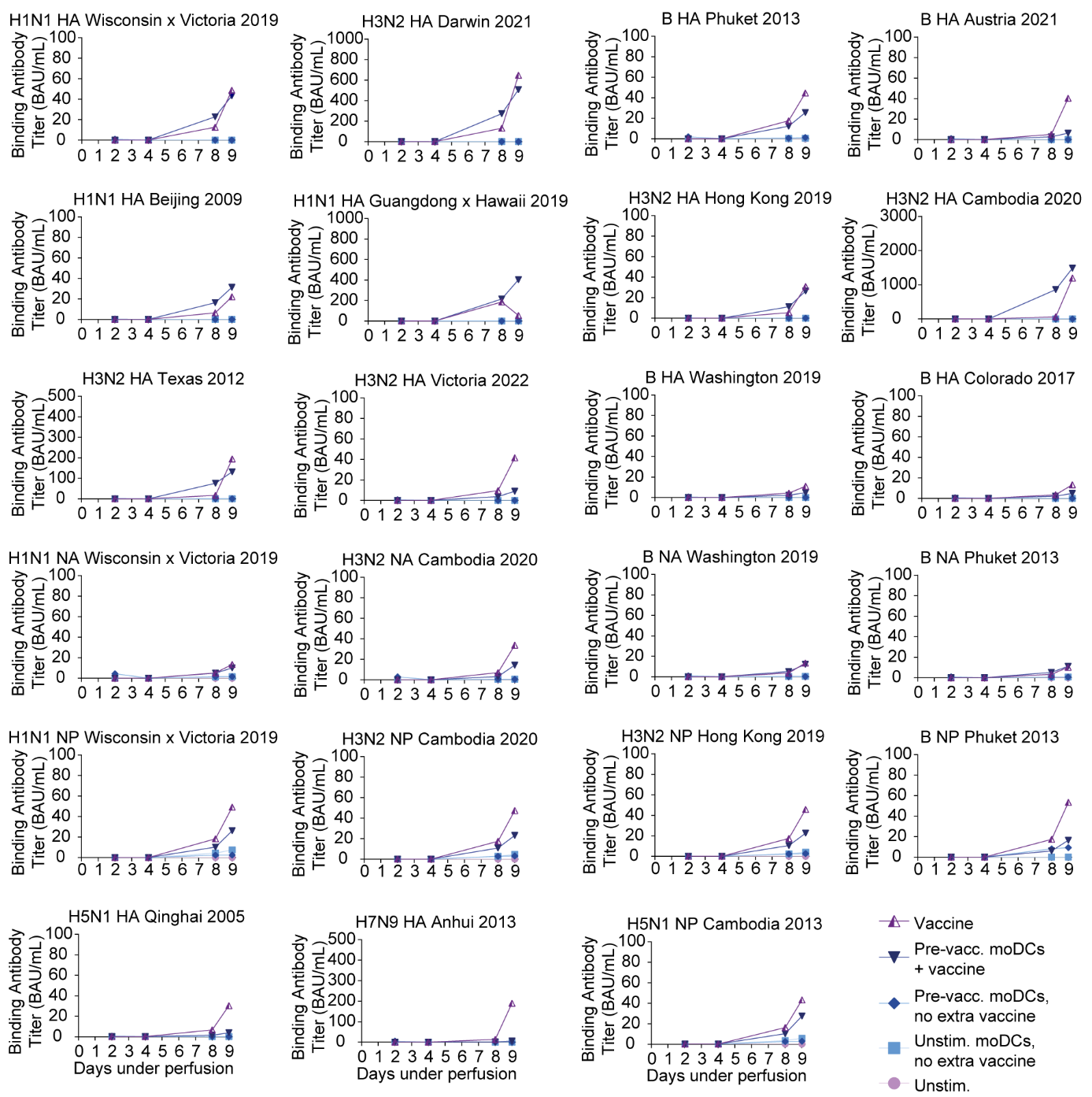


**Figure S9.** Impact of mode of antigen delivery on influenza antigen-specificity. LToCs were generated, perfused with medium for two days and then vaccinated overnight by supplementing perfused medium with either vaccine (vaccine D2), non-vaccinated moDCs (unstim. moDCs, no extra vaccine), moDCs pre-vaccinated for three hours (pre-vacc. moDCs, no extra vaccine), both pre-vaccinated moDCs and vaccine (pre-vacc. moDCs + vaccine) or no vaccine (unstim.). Multiplex hemagglutinin subtype-specific antibody detection was applied to detect binding antibody units (BAU) per ml of antibodies against different influenza strains in effluents from donor 4. n = 2 chips per condition (Donor 5), standard deviation not shown.

**Table S1. Patient metrics.**

Information on sex and age of tonsil donor.

| **Donor** | **Sex** | **Age** | **Reason for surgery** |
| --- | --- | --- | --- |
| 1 (0016) | female | 19 | Recurrent tonsillitis |
| 2 (0017) | female | 20 | Recurrent tonsillitis |
| 3 (0061) | female | 27 | Recurrent tonsillitis |
| 4 (0010) | female | 21 | Recurrent tonsillitis |
| 5 (0038) | female | 30 | Recurrent tonsillitis |
| 6 (0055) | male | 52 | Recurrent tonsillitis, recurrent tonsilloliths |

**Table S2. List of cytokines included in assay to assess chip ad-/absorption.**

| Analyte | Lymphoid tissue-associated? | Lower limit of quantification (pg/ml) | Upper limit of quantification (pg/ml) |
| --- | --- | --- | --- |
| IL-1-β/IL-1F2 | Yes | 7.57 | 5,520.00 |
| TNF-α | Yes | 3.06 | 2,230.00 |
| CCL7/MCP-3/MARC | Yes | 19.60 | 14,290.00 |
| IL-6R-α | Yes | 92.89 | 67,720.00 |
| CCL18/PARC | Yes | 6.23 | 4,540.00 |
| CCL19/MIP-3-β | Yes | 3.16 | 2,300.00 |
| BAFF/BLyS/TNFSF13B | Yes | 5.76 | 4,200.00 |
| CX3CL1/Fractalkine | Yes | 592.06 | 143,870.00 |
| CCL23/MPIF-1 | Yes | 38.02 | 9,240.00 |
| Granzyme B | Yes | 15.64 | 3,800.00 |
| CD30/TNFRSF8 | Yes | 1.69 | 410.00 |
| IFN-α | Yes | 3.07 | 2,240.00 |
| Chemerin | Yes | 411.81 | 100,070.00 |
| IL-6 | Yes | 4.12 | 1,000.00 |
| Fas/TNFRSF6/CD95 | Yes | 43.10 | 31,420.00 |
| CCL5/RANTES | Yes | 6.43 | 4,690.00 |
| 4-1BB/TNFRSF9/CD137 | Yes | 9.34 | 6,810.00 |
| CCL1/I-309/TCA-3 | Yes | 1.88 | 1,370.00 |
| CXCL10/IP-10/CRG-2 | Yes | 0.52 | 380.00 |
| IFN-β | Yes | 13.91 | 3,380.00 |
| IL-10 | Yes | 1.39 | 1,010.00 |
| CCL2/JE/MCP-1 | Yes | 29.67 | 7,210.00 |
| IFN-gamma | Yes | 3.13 | 760.00 |
| CCL4/MIP-1-β | Yes | 123.42 | 29,990.00 |
| IL-8/CXCL8 | Yes | 1.65 | 1,200.00 |
| Aggrecan | Yes | 306.01 | 74,360.00 |
| CXCL1/GRO-α /KC/CINC-1 | Yes | 168.77 | 13,670.00 |

**Table S3. List of analytes included within cytokine assay as well as their specific mixes for LToC effluent analysis.**

Analytes were split into 4 bead mixes to avoid cross-reactivity.

| Bead Mix | Analyte | Lymphoid tissue-associated? | Lower limit of quantification (pg/ml) | Upper limit of quantification (pg/ml) | Samples within detection limit? |
| --- | --- | --- | --- | --- | --- |
| 1 | IL-1-β/IL-1F2 | Yes | 6.35 | 4,630.00 | Below |
| 1 | IL-4 | Yes | 5.06 | 3,690.00 | Below |
| 1 | IL-2 | Yes | 13.29 | 9,690.00 | Below |
| 1 | G-CSF | Yes | 7.90 | 5,760.00 | Below |
| 1 | MBL | Yes | 15.34 | 11,180.00 | Below |
| 1 | CCL17/TARC | Yes | 32.24 | 23,500.00 | Yes |
| 1 | IL-12/IL-23 p40 | Yes | 163.83 | 119,430.00 | Below |
| 2 | BDNF | No | 9.25 | 6,740.00 | Below |
| 2 | MIF | Yes | 177.00 | 128,850.00 | Yes |
| 2 | IL-27 | Yes | 269.09 | 65,390.00 | Below |
| 2 | Pentraxin 3/TSG-14 | Yes | 52.50 | 38,361.00 | Below |
| 2 | IL-17E/IL-25 | Yes | 85.80 | 62,651.00 | Below |
| 2 | Prolactin | No | 385.54 | 281,060.00 | Below |
| 2 | Lymphotoxin-α/TNF-β | Yes | 2.02 | 1,470.00 | Yes |
| 2 | IL-13 | Yes | 156.97 | 114,430.00 | Below |
| 2 | M-CSF | No | 43.60 | 31,841.00 | Yes |
| 2 | Myeloperoxidase/MPO | No | 80.12 | 19,470.00 | Yes |
| 2 | FGF-23 | No | 2.29 | 1,670.00 | Below |
| 2 | RBP4/Retinol Binding Protein 4 | No | 1,284.00 | 104,641.00 | Medium level |
| 2 | CXCL11/I-TAC | Yes | 7.43 | 5,420.00 | Below |
| 2 | FGF acidic/FGF1 | No | 17.49 | 4,250.00 | Below |
| 2 | BCMA/TNFRSF17 | Yes | 9.25 | 6,740.00 | Yes |
| 2 | TIMP-1 | No | 16.47 | 12,010.00 | Yes |
| 3 | TNF-α | Yes | 2.21 | 1,610.00 | Yes |
| 3 | gp130 | Yes | 122.46 | 89,270.00 | Yes |
| 3 | IL-3 | Yes | 27.64 | 20,150.00 | Yes |
| 3 | CCL7/MCP-3/MARC | Yes | 14.69 | 10,710.00 | Yes |
| 3 | IL-6R-α | Yes | 79.82 | 58,190.00 | Yes |
| 3 | CXCL13/BLC/BCA-1 | Yes | 5.28 | 3,850.00 | Yes |
| 3 | CCL18/PARC | Yes | 5.38 | 3,920.00 | Yes |
| 3 | CCL3/MIP-1-α | Yes | 104.90 | 25,490.00 | Yes |
| 3 | IL-31 | Yes | 27.94 | 20,370.00 | Yes |
| 3 | CCL8/MCP-2 | Yes | 14.69 | 3,570.00 | Yes |
| 3 | CCL19/MIP-3-β | Yes | 2.67 | 1,950.00 | Yes |
| 3 | BAFF/BLyS/TNFSF13B | Yes | 7.19 | 5,240.00 | Above |
| 3 | CX3CL1/Fractalkine | Yes | 474.20 | 115,230.00 | Yes |
| 3 | Complement Component C9 | Yes | 6,419.27 | 4,679,650.00 | Yes |
| 3 | Resistin | No | 39.47 | 9,590.00 | Below |
| 3 | IL-15 | Yes | 6.13 | 1,490.00 | Medium level |
| 3 | Collagen IV α1 | Yes | 22.00 | 15,960.00 | Yes |
| 3 | CCL23/MPIF-1 | Yes | 21.89 | 15,960.00 | Yes |
| 3 | Granzyme B | Yes | 10.91 | 7,950.00 | Yes |
| 3 | CD30/TNFRSF8 | Yes | 0.70 | 510.00 | Yes |
| 3 | IFN-α | Yes | 3.70 | 2,700.00 | Yes |
| 3 | Chemerin | Yes | 471.98 | 114,690.00 | Medium level |
| 3 | IL-6 | Yes | 1.00 | 1,191.00 | Yes |
| 3 | Fas/TNFRSF6/CD95 | Yes | 1.63 | 1,190.00 | Yes |
| 3 | CCL5/RANTES | Yes | 8.16 | 5,950.00 | Yes |
| 3 | CCL11/Eotaxin | Yes | 22.94 | 16,720.00 | Yes |
| 4 | IL-28A/IFN-lambda 2 | Yes | 50.86 | 37,080.00 | Below |
| 4 | 4-1BB/TNFRSF9/CD137 | Yes | 8.20 | 5,980.00 | Yes |
| 4 | IL-33 | Yes | 15.76 | 3,830.00 | Below |
| 4 | CCL1/I-309/TCA-3 | Yes | 1.39 | 1,010.00 | Yes |
| 4 | Complement Component C5a | Yes | 1026.50 | 249,440.00 | Below |
| 4 | DPPIV/CD26 | No | 200.78 | 48,790.00 | Medium level |
| 4 | IL-7 | Yes | 1.67 | 1,220.00 | Below |
| 4 | IFN-β | Yes | 4.72 | 3,440.00 | Below |
| 4 | IL-10 | Yes | 1.67 | 1,220.00 | Yes |
| 4 | CCL2/JE/MCP-1 | Yes | 32.43 | 7,881.00 | Below |
| 4 | Angiopoietin-2 | No | 28.66 | 20,890.00 | Below |
| 4 | CXCL2/GRO-β/MIP-2/CINC-3 | Yes | 17.12 | 12,480.00 | Below |
| 4 | CCL14/HCC-1/HCC-3 | Yes | 44.02 | 32,090.00 | Below |
| 4 | IFN-gamma | Yes | 2.39 | 580.00 | Yes |
| 4 | IL-1ra/IL-1F3 | Yes | 32.18 | 7,820.00 | Yes |
| 4 | CCL20/MIP-3-α | Yes | 6.87 | 1,670.00 | Below |
| 4 | HGF | No | 17.41 | 4,230.00 | Below |
| 4 | CCL15/MIP-1 delta | Yes | 842.72 | 204,780.00 | Below |
| 4 | CCL4/MIP-1-β | Yes | 151.73 | 36,870.00 | Below |
| 4 | IL-1-α/IL-1F1 | No | 4.69 | 1,140.00 | Below |
| 4 | IL-17/IL-17A | Yes | 15.39 | 3,740.00 | Yes |
| 4 | APRIL/TNFSF13 | Yes | 97.33 | 23,650.00 | Below |
| 4 | Adiponectin/Acrp30 | No | 877.00 | 213,110.00 | Below |
| 4 | GM-CSF | Yes | 39.88 | 3,230.00 | Below |
| 4 | FGF basic/FGF2/Bfgf | No | 1.48 | 360.00 | Medium level |
| 4 | IL-8/CXCL8 | Yes | 2.15 | 1,570.00 | Yes |
| 4 | IL-5 | Yes | 6.67 | 1,620.00 | Below |
| 4 | IL-12 p70 | Yes | 50.14 | 36,550.00 | Below |
| 4 | VCAM-1/CD106 | Yes | 3,089.95 | 2,252,570.00 | Yes |
| 4 | VEGF-A | No | 3.83 | 2,790.00 | Yes |
| 4 | Complement Factor D/Adipsin | No | 267.34 | 194,890.00 | Below |
| 4 | IL-21 | Yes | 9.19 | 6,700.00 | Below |
| 4 | α-Fetoprotein/AFP | No | 260.03 | 189,560.00 | Below |
| 4 | BMP-2 | No | 48.64 | 3,940.00 | Below |
| 4 | BMP-4 | No | 6.94 | 5,060.00 | Below |
| 4 | BMP-9 | No | 0.74 | 540.00 | Below |
| 4 | Serpin A12 | No | 17.13 | 12,490.00 | Below |
| 4 | Aggrecan | Yes | 328.89 | 79,920.00 | Medium level |
| 4 | IL-23 | Yes | 53.58 | 39,060.00 | Yes |
| 4 | CXCL1/GRO-α /KC/CINC-1 | Yes | 160.49 | 13,000.00 | Below |
| 4 | IL-18/IL-1F4 | Yes | 41.73 | 3,380.00 | Below |

**Table S4. Antigens included within Influenza multiplex binding assay.**

| Subtype | Protein | Strain | Manufacturer | Catalogue # |
| --- | --- | --- | --- | --- |
| H1N1 | HA | Wisconsin x Victoria 2019 | Sino Biological | 40787-V08H |
| H1N1 | HA | Beijing 2009 | Sino Biological | 40035-V08H |
| H1N1 | HA | Victoria 2022 | Sino Biological | 40938-V08H |
| H1N1 | HA | Guangdong x Hawaii 2019 | Sino Biological | 40717-V08H |
| H3N2 | HA | Cambodia 2020 | Sino Biological | 40789-V08H |
| H3N2 | HA | Hong Kong 2019 | Sino Biological | 40721-V08H |
| H3N2 | HA | Darwin 2021 | Sino Biological | 40859-V08H |
| H3N2 | HA | Texas 2012 | Sino Biological | 40354-V08H1 |
| Yamagata | HA | Phuket 2013 | Sino Biological | 40498-V08H1 |
| Victoria | HA | Austria 2021 | Sino Biological | 40862-V08H |
| Victoria | HA | Colorado 2017 | Sino Biological | 40581-V08H |
| Victoria | HA | Washington 2019 | Sino Biological | 40722-V08H |
| H7N9 | HA | Anhui 2013 | Sino Biological | 40103-V08H |
| H5N1 | HA | Qinghai 2005 | Sino Biological | 40117-V08B |
| H1N1 | NA | Wisconsin x Victoria 2019 | Sino Biological | 40785-V08B |
| H3N2 | NA | Cambodia 2020 | Sino Biological | 40784-V08B |
| Yamagata | NA | Phuket 2013 | Sino Biological | 40502-V07B |
| Victoria | NA | Washington 2019 | Sino Biological | 40790-V08B |
| H1N1 | NP | Wisconsin x Victoria 2019 | Sino Biological | 40774-V08B |
| H3N2 | NP | Cambodia 2020 | Sino Biological | 40778-V08B |
| H3N2 | NP | Hong Kong 2019 | Sino Biological | 40753-V08B |
| Yamagata | NP | Phuket 2013 | Sino Biological | 40500-V08B |
| H5N1 | NP | Cambodia 2013 | Sino Biological | 40947-V08B |
